# Astrocytic Extracellular Vesicles Regulated by Microglial Inflammatory Responses Improve Stroke Recovery

**DOI:** 10.1007/s12035-023-03629-9

**Published:** 2023-09-07

**Authors:** Chikage Kijima, Toshiki Inaba, Kenichiro Hira, Nobukazu Miyamoto, Kazuo Yamashiro, Takao Urabe, Nobutaka Hattori, Yuji Ueno

**Affiliations:** 1 Department of Neurology, Juntendo University Faculty of Medicine, Tokyo, Japan; 2 Department of Neurology, Juntendo University Urayasu Hospital, Chiba, Japan; 3 Neurodegenerative Disorders Collaborative Laboratory, RIKEN Center for Brain Science, Saitama, Japan

**Keywords:** Stroke, Reactive astrocytes, Microglia, P2Y_1_ receptor, Extracellular vesicles

## Abstract

There are no effective treatments for post-stroke glial scar formation, which inhibits axonal outgrowth and functional recovery after stroke. We investigated whether astrocytic extracellular vesicles (AEVs) regulated by microglia modulate glial scars and improve stroke recovery. We found that peri-infarct glial scars comprised reactive astrocytes with proliferating C3d and decreased S100A10 expression in chronic stroke. In cultured astrocytes, microglia-conditioned media and treatment with P2Y_1_ receptor antagonists increased and reduced the area of S100A10- and C3d-expressing reactive astrocytes, respectively, by suppressing mitogen-activated protein kinase/nuclear factor-κβ (NF-κB)/tumor necrosis factor-α (TNF-α)/interleukin-1β signaling after oxygen–glucose deprivation. Intracerebral administrations of AEVs enriched miR-146a-5p, downregulated NF-κB, and suppressed TNF-α expressions, by transforming reactive astrocytes to those with S100A10 preponderance, causing functional recovery in rats subjected to middle cerebral artery occlusion. Modulating neuroinflammation in post-stroke glial scars could permit axonal outgrowth, thus providing a basis for stroke recovery with neuroprotective AEVs.

**Supplementary Information:**

The online version contains supplementary material available at 10.1007/s12035-023-03629-9.

## Introduction

Stroke is the leading cause of severe disability worldwide. The benefits of conventional rehabilitation therapy are limited in improving functional recovery after stroke, and regenerative therapies, such as mesenchymal stem/stromal cell (MSC) transplantation, have been challenging. There are several issues related to the application of cell therapies in clinical practice, including tumor formation, high costs, and low grafting rates [1, 2]. Thus, there is an urgent need to develop alternative therapies that facilitate functional recovery based on neuroregeneration, such as that associated with axonal outgrowth.

Astrocytes and microglia are activated under the pathological conditions of central nervous system (CNS). Astrocytes are crucial in reducing brain damage, rebuilding the blood–brain barrier, and maintaining CNS homeostasis in the acute stage of ischemia [3, 4]. In the chronic stage, however, astrocytes form glial scars and hinder axonal regeneration in the peri-infarct area [5]. On the contrary, emerging data indicate that glial scars that produce chondroitin sulfate proteoglycan 4 (*cspg4*) and *cspg5* are essential for axonal regeneration after spinal cord injury [6]. In 2017, Liddelow et al. revealed that reactive astrocytes had binary phenotypes (neurotoxic, A1; neuroprotective, A2). However, many transcriptomic studies indicate the diversity of reactive astrocytes with distinct molecular states in different disease models and regions in the CNS rather than this binary polarization [7]. Regarding post-ischemic reactive astrocytes, differential expression profiles of genes have been identified [8–10].

There has been growing insight into the crosstalk between microglia and astrocytes, exerting neuroprotective astrocytes [11]. Particularly, the inflammatory mechanistic linkage between microglia and astrocytes is crucial; however, the modulation of this interaction for chronic glial scar formation has not been fully elucidated. The P2Y_1_ receptor (P2Y_1_R) in the astrocytes is inhibited by cytokines from the activated microglia after traumatic brain injury, resulting in the induction of neuroprotective astrocytes [11]. Thus, it is suggested that the anti-inflammatory modulation of reactive astrocytes by microglial regulation and an inhibition of the P2Y_1_R expression may be related to functional recovery in the chronic stage of stroke.

Extracellular vesicles (EVs), including exosomes, enriched in microRNAs (miRNAs), mRNAs, nucleic acids, lipids, and proteins including tetraspanins, are involved in intercellular communication in the CNS under both normal physiological and pathologic conditions, especially in the crosstalk between glial cells and neurons [12, 13]. EV treatment is superior to cell therapy as it involves less tumorigenicity, no occlusion of the microvascular system, low immunogenicity that does not require a host immune response, and tough lipid bilayer vesicles that retain bioactivity [13, 14]. EVs derived from MSCs for the treatment of stroke display the same tissue regeneration capability as MSCs themselves, and treatment with such EVs not only enhances neurogenesis, angiogenesis, and axonal outgrowth but also suppresses inflammatory reactions [15, 16]. Previously, we found that changes in a subset of miRNAs in astrocyte-derived EVs (AEVs) with the downregulation of miR-30c-2-3p and miR-326-5p expressions facilitated axonal regeneration and functional recovery after stroke [12]. To date, an increasing number of studies have found the efficacy of MSC-derived EVs on restorative therapy in stroke. Although treatment of AEVs can be essentially physiological for brain tissue, the evidence of AEVs for stroke recovery is limited.

Here, we aimed to develop a therapy to reduce the sequelae by regulating post-stroke glial scars. It is hypothesized that neuroinflammation can be implicated in glial scar formation, which integrates to hinder or enhance axonal outgrowth in the chronic stage of stroke. We analyzed the molecular mechanisms of ischemic astrocytes against neuroinflammation regulated by microglia or inhibition of P2Y_1_R and the therapeutic effects of these ischemic astrocyte-derived EVs to modulate glial scars and facilitate recovery from stroke.

## Materials and methods

All experimental procedures were approved by the Animal Care Committee of the Juntendo University (No. 2,021,116).

### Focal Cerebral Ischemia

Adult male Wistar/ST rats (8–10 weeks old, approximately 320 g, Charles River, Kanagawa, Japan) were used in this study. Rats were anesthetized with 4.0% isoflurane (Abbott Japan CO., LTD, Tokyo, Japan), maintained in 1.0–1.5% isoflurane in 70% N_2_O and 30% O_2_, and were subjected to permanent right middle cerebral artery occlusion (MCAO) by advancing a 4 to 0 surgical nylon suture with an expanded tip from the internal carotid artery [12]. At 3, 7, 14, 21, 28, and 56 days after MCAO, the rats were deeply anesthetized with pentobarbital and perfused transcardially. The brain was removed immediately, post-fixed for 24 h in 4% paraformaldehyde, and soaked in 30% sucrose for at least 48 h. For histological and immunohistochemical studies, 20-µm thick coronal sections of the brain were prepared using a cryostat (Leica Biosystems, Wetzlar, Germany).

### Behavioral Analyses

To assess behavioral functions after stroke, we screened for neurological deficits using the modified neurological severity scores (mNSSs) and further examined motor function using the rotarod test when the mNSS exhibited a significant difference. The mNSS is a composite of the motor, sensory, reflex, and balance tests [17]. The mNSS was graded on a scale of 0 to 18 (normal score, 0; total score, 18). The higher the mNSS score, the more serious the neurological damage. The mNSS was examined 7, 14, 21, 28, and 56 days after MCAO in each treatment group. The survival rates and body weights were also evaluated. For the rotarod test, rats were placed on a rotarod cylinder (MK-660D; Muromachi Kikai Co., Ltd., Tokyo, Japan), and the time the rats remained on the rotarod was recorded [12]. The speed was slowly increased from 10 to 40 rpm (0.005–0.08 *g*) over 2 min. Six rotarod measurements were conducted, and the average of the shortest, second shortest, and longest latencies to fall off the rotarod were calculated and compared among the groups.

### Primary Cultures

Primary microglia/astrocyte cultures were prepared from the cerebral cortices of 0- to 2-day-old neonatal Sprague–Dawley rats (Oriental Yeast Co., Ltd.) according to a previously published protocol with minor modifications [12]. Dissociated cells were plated in 75-cm^2^ cell culture flasks and maintained in Dulbecco’s Modified Eagle Medium (DMEM) (Life Technologies, Washington, DC) containing 25 mM glucose, 4 mM glutamine, 1 mM sodium pyruvate, 20% heat-inactivated FBS, and 1% penicillin/streptomycin for approximately 10 days until confluency. To collect microglia, flasks were shaken for 1 h on an orbital shaker (200 rpm) at 37 °C. The medium was re-plated onto poly-DL-ornithine-coated plates in DMEM containing 10% exosome depleted heat-inactivated FBS and 1% antibiotic-antimycotic (Life Technologies, Japan, Tokyo). After 4–5 days, medium was changed to Ca^2+^-and Mg^2+^ free Hanks balanced salt solution, and cultured microglia were challenged with oxygen–glucose deprivation (OGD) (5% CO_2_/95% N_2_ incubator for 3 h). The medium was changed to DMEM containing 10% exosome depleted heat-inactivated FBS and 1% antibiotic-antimycotic and incubated for 96 h. After collecting microglia, the flasks were replaced with fresh medium and shaken overnight to remove oligoprogenitor cells. Adherent astrocytes were dissociated by trypsinization and then replaced in cell culture 75-cm^2^ flasks. Seven hours after replacement, the flasks were shaken overnight, and the medium was changed to collect the astrocytes. After overnight replacement, astrocytes were trypsinized onto poly DL-ornithine-coated plates. Cells were challenged with OGD for 3 h after reaching 70–80% confluency. After OGD, the medium was changed to DMEM containing 2% B27 and 1% penicillin/streptomycin and incubated for 96 h. Ten to 12 neonatal rats per experiment were gathered and prepared for primary astrocyte culture, and each experiment was counted as the number in vitro.

### Primary Cortical Neurons

Cortical cells were harvested from embryonic day 17 Wistar rats (Charles River) according to a published protocol with minor modifications [18]. In brief, dissociated cortical cells at a density of 3 × 10^7^ cells/mL were cultured in microfluidic chambers (AXIS™ Axon Isolation Device, Millipore, Billerica, MA) in neurobasal medium with 2% B27, which included serum albumin, corticosterone, insulin, and progesterone (GIBCO, Grand Island, NY). Uridine and 5-fluorodeoxyuridine were added for 6 days to kill astrocytes. After 7 days in vitro, the medium was changed to Ca2^+^- and Mg2^+^-free Hanks’ balanced salt solution, and cultured neurons were challenged with OGD for 3 h. After OGD, cultured neurons were incubated in neurobasal medium containing 2% B27 for 96 h. Ten to 12 embryos were gathered and prepared for primary neuronal culture, and each experiment was counted as the number in vitro.

### Isolation of AEVs from Cultured Astrocytes

After removing the cells and cell debris by centrifugation at 3000 × *g* for 15 min, the astrocyte culture medium and ExoQuick-TC Exosome Precipitation Solution (System Bioscience, Mountain View, CA) were mixed in a 5:1 ratio. The mixtures were incubated overnight at 4 °C and centrifuged at 1500 × *g* for 30 min. AEVs were obtained as precipitates. The supernatant was removed by careful aspiration. To analyze the protein concentration of AEVs, supernatants were dissolved in PBS, and a Micro BCA protein assay kit (Thermo Scientific, Waltham, MA) was used.

### Treatment with P2Y_1_R Antagonist (P2Y_1_R-ANT) of Primary Cultured Microglia and Astrocytes

In cultured microglia, 1 mmol/l P2Y_1_R-ANT (Sigma–Aldrich, Saint Louis, MO) was randomly administered after OGD and microglia were incubated for 96 h. Cultured astrocytes were randomly allocated to the following experimental groups: (1) non-OGD, (2) OGD, (3) OGD and treatment with microglia-conditioned media (MCM), (4) OGD and treatment with 1 mmol/l P2Y_1_R-ANT, or (5) OGD and treatment with 1 mmol/l P2Y_1_R-ANT and MCM that was gathered from incubation with ischemic microglia at 96 h after OGD. P2Y_1_R-ANT and MCM were administered immediately administered after OGD, and astrocytes were incubated for 96 h. Cultured astrocytes and astrocyte-conditioned media were collected 96 h after OGD for analyses (N = 4–5/group).

### Application of AEVs and Tumor Necrosis factor-α (TNF-α) to Cultured Primary Neurons

Cultured cortical neurons challenged with OGD were randomly allocated to the following experimental groups: (1) no treatment, (2) AEVs derived from OGD astrocytes, and (3) AEVs derived from OGD astrocytes treated with 1 mmol/l P2Y_1_R-ANT and MCM. We placed 3 × 10^9^ AEVs into the somal compartment and 3 × 10^8^ AEVs into the axonal compartment according to a previously published protocol [12]. Cultured cortical neurons after OGD were also randomly allocated to the following experimental groups: (1) no treatment, (2) conditioned with 1 ng/µl recombinant TNF-α (BioLegend, San Diego, CA, USA), or (3) conditioned with 10 ng/µl recombinant TNF-α (N = 4–5/group). AEVs and recombinant TNF-α were administered immediately after OGD, and neurons were incubated with such treatments for 96 h.

### Time-lapse Microscopic Analysis

Time-lapse images to track axonal elongation in primary neuronal cultures grown in microfluidic chambers were obtained using a BioStation CT incubator (Nikon, Tokyo, Japan) equipped with a camera for video imaging. Under bright field conditions, axonal elongation after OGD was measured every 30 min for 6 h using a 20× lens. Four to six individual distal axons per chamber were randomly selected.

### Intracerebral Administration of P2Y_1_-R-ANT and AEVs

Using an osmotic minipump (Alzet Corporation, type 1004, Cupertino, CA, USA), selective P2Y_1_R-ANT (1 mM, 10 mM, Tocris Bioscience, Bristol, UK) was administered into the peri-infarct area (0.45 mm anterior to bregma, 3.0 mm lateral to midline, and 2 mm deep from the brain surface) for 14 days from 7 to 21 days after MCAO. AEVs were isolated from the medium in cultured astrocytes 96 h after OGD and 96 h after OGD with treatment with P2Y_1_R-ANT and MCM. Astrocyte-derived AEVs (100 µg) were once administered into the peri-infarct area at 7 days after MCAO using a micro syringe (Ito Corporation, Shizuoka, JAPAN). In the control group, PBS was used as the vehicle-treated group.

### Immunohistochemistry and Immunocytochemistry

Immunofluorescence staining was performed as previously described [12, 18]. After washing with PBS, rat coronal brain sections were blocked with 2% Block Ace (Dainippon Sumitomo Pharma, Osaka, Japan) for 1 h and then incubated with primary antibodies overnight at 4 ℃. The primary antibodies used in this study were rabbit polyclonal anti-ionized calcium-binding adapter molecule 1 (Iba-1) (1:800; Wako), mouse monoclonal anti-glial fibrillary acidic protein (GFAP) (1:200; MBL), goat polyclonal anti-GFAP (1:200; Abcam), goat polyclonal anti-C3d (1:25, R&D Systems), rabbit polyclonal anti-S100A10 (1:50, Proteintech), rabbit polyclonal anti-P2Y_1_ receptor antibody (1:100, Alomone Labs), rabbit polyclonal anti-nuclear factor-κβ (NF-κB) (1:200; Abcam), mouse monoclonal anti-TNF-α (1:200; GeneTex), mouse monoclonal pNFH (SMI31)(1:200; BioLegend), and mouse MAP2 (1:500; Merck). After washing with PBS, the sections were incubated with secondary antibodies (1:250; Jackson ImmunoResearch Laboratories) for 1 h at room temperature, and the slides were covered with Vectashield mounting medium with 4’,6-diamidino-2-phenylindole (Vector Laboratories). Primary microglia on cover glasses in culture dishes were washed with PBS and fixed with 4% paraformaldehyde for 15 min. After washing with 0.3% Tryton X-100 (Bio-Rad Laboratories), cells were blocked with 2% Block Ace in PBS for 1 h. The primary antibodies used were rabbit polyclonal anti-Iba-1(1:800; Wako), goat polyclonal anti-liver arginase (1:50, Abcam), and mouse monoclonal anti-inducible nitric oxide synthase (iNOS) (1:300; Abcam). After washing with PBS, the sections were incubated with secondary antibodies (1:200; Jackson ImmunoResearch Laboratories) for 1 h at room temperature, and the slides were covered with Vectashield.

### Western blot

Cultured astrocytes and neurons were collected in lysis buffer (Cell Lytic Mammalian Tissue Lysis/Extraction Reagent, Sigma–Aldrich) and protein extraction and electrophoresis were performed. After electrophoresis and transfer to polyvinylidene difluoride membranes, the membranes were blocked with 1% bovine serum albumin in Tween PBS for 60 min. The membranes were then incubated overnight at 4 °C with primary antibodies. The primary antibodies used in this study were goat polyclonal anti-GFAP (1:40,000; Abcam), goat polyclonal anti-C3d (a marker for A1 astrocytes) (1:2,000, R&D Systems), rabbit polyclonal anti-S100A10 (1:2,000, Proteintech), mouse monoclonal anti-CSPG (1:100,000, Abcam), mouse monoclonal anti-phosphorylated neurofilament heavy chain (pNFH) (1:500; BioLegend), rabbit polyclonal anti-P2Y_1_R-ANT (1:200; Alomone Labs), rabbit polyclonal anti-caspase-3 (1:500, Abcam), and rabbit monoclonal anti-actin (1:10,000, Abcam). The membranes were incubated with peroxidase-conjugated secondary antibodies (1:5,000; Santa Cruz Biotechnology), and the bound proteins were visualized using enhanced chemiluminescence (GE Healthcare). Protein expression was analyzed by densitometry, which evaluates the relative amount of protein staining and quantifies the results in terms of optical density using ImageJ software (v1.53k). Two independent samples were used per experimental condition (N = 4–5). The optical density of each protein band was normalized to that of β-actin. Arbitrary units of relative expression in the treatment groups were divided into those in the OGD group.

### Cell Viability Assays

Astrocyte viability was measured using the Cell Counting Kit 8 (CCK-8) cytotoxicity assay (Dojindo Molecular Technologies, Inc., Kumamoto, Japan), which allows convenient assessment with highly water-soluble tetrazolium salt. Astrocytes seeded in a six-well plate were challenged with OGD for 3 h, and the medium was replaced with 2000 µL complete medium containing 100 µL CCK-8 solution. The plate was incubated for 96 h at 37 °C. The absorbance at 450 nm was measured using an iMark microplate reader (Bio-Rad, Hercules, CA).

### Evaluation for TNF-α and interleukin‐1β (IL‐1β) on enzyme‐linked Immunosorbent Assay (ELISA)

TNF-α (R&D Systems; minimum detectable dose 1.88 pg/mL), and IL‐1β (R&D Systems, Minneapolis, MN; minimum detectable dose 2.31 pg/mL) on astrocyte-conditioned media were measured using the ELISA kits according to the manufacturer’s protocol. Optical density was measured at 450 nm minus those obtained at 570 nm using an ELISA reader (Bio‐Rad Laboratories, Inc., Hercules, CA). The concentrations of IL‐1β and TNF‐α were obtained according to the standard curve.

### Microglial Morphology Analysis

In microglial cultures, two dishes in each experiment were prepared. In 1 dish, 30 to 60 Iba-1^+^ cells were counted as previously described [19, 20]. Morphology of microglia such as resting, bipolar/rod-shaped, and amoeboid types were defined as having short but sturdy, multiple processes; rod-shaped or flat without thin processes; and polarized microglia with thin processes, respectively.

### Transmission electron Microscopy

A droplet of AEV samples (10 µL) was pipetted onto a carbon-film grid for 10 s, on which excess liquid was blotted off by touching one end of the grid with filter paper. After the grid was partially dried, 20 µL drop of 2% (w/v) uranyl acetate was placed for 10 s, and the excess liquid was blotted off with filter paper. The grids were dried at room temperature and visualized using a JEOL JEM1400 Flash electron microscope (JEOL, Tokyo, Japan) at 100 k.

### Analysis of size Distribution and Tetraspanin Expression of AEVs

To analyze the size distribution of AEVs, nano-tracking analysis was performed using the NanoSight platform (NanoSight LM10, Malvern Panalytical, Kassel, Germany). According to a previous study, with minor modifications [21], 1:2000 PBS-diluted samples were measured in duplicate, and 400 µl of the diluted sample was injected into the measurement chamber. Each sample was measured five times, and the length of the video for each measurement was set to 30 s. ExoView software was used according to the manufacturer’s instructions.

To analyze tetraspanin expression, AEVs were diluted in a specified solution supplied in the ExoView kit at a concentration of 10^10^ particle/ml. Diluted samples (35 µL) were placed onto the kit microarray chips equipped with capture antibodies for CD63 (NanoView Biosciences), CD81 (NanoView Biosciences), CD9 (BioLegend), and negative control IgG (NanoView Biosciences) in a 24-well plate, sealed, and incubated for 24 h at room temperature. After incubation, 1000 µl of the solution was added to each well. The plate was placed on an orbital shaker at 500 rpm for 3 min, and 750 µl of the solution was removed from each well. After washing three times, the microarray chips were incubated with anti-CD81 Alexa-555, anti-CD63 Alexa-488, and anti-CD9 Alexa-647 dissolved in blocking solution on an orbital shaker at 500 rpm shielded from light with aluminum foil. After 1 h of incubation, 500 µl of the solution was added to each well, and 750 µl of the solution was removed from each well. The plate was then placed on an orbital shaker at 500 rpm for 3 min. The same process was repeated three times using other solutions, ending with the addition of deionized water (DI) water and shaking in the same manner. The chips were rinsed with fresh DI water in a 10 cm dish and placed on an absorbent paper. The chips were analyzed using ExoView R100 IMAGER (NanoView Biosciences, USA) with ExoScan software. The data were then analyzed using ExoViewer 2.7.9 (N = 3).

### Microarray data Analysis of mRNAs and miRNA

Total RNA was isolated from cultured astrocytes using a RNeasy Mini Kit (Qiagen, Hilden, Germany). Microarray analysis of astrocytic mRNAs was performed at Hokkaido System Science (Sapporo, Japan) using the SurePrint G3 Rat GE 8 × 60 K ver. 2.0 (Agilent Technologies, Santa Clara, CA, USA) according to the manufacturer’s protocol. Differentially expressed genes were identified among the following groups: cultured astrocytes challenged to OGD vs. cultured astrocytes challenged to OGD and treatment with P2Y_1_R-ANT; cultured astrocytes challenged to OGD and treatment with P2Y_1_R-ANT vs. cultured astrocytes challenged to OGD and treatment with P2Y_1_R-ANT and MCM, and cultured astrocytes challenged to OGD and treatment with P2Y_1_R-ANT and MCM vs. cultured astrocytes challenged to OGD. All groups were analyzed and functionally categorized, and their interactions were clarified using Ingenuity Pathway Analysis (IPA) software (Ingenuity Systems, Redwood City, CA, USA). The miRNAs were isolated from AEVs using the miRNeasy Micro Kit (Qiagen). A microarray analysis of miRNAs from AEVs was performed at Hokkaido System Science using Rat miRNA 8 × 15 K Rel. 21.0 (Agilent Technologies). For canonical pathway analysis, the − log (P-value) > 2 was taken as threshold; a z-score > 2 and < − 2 was defined as the threshold of significant activation and inhibition, respectively. For regulatory effects and molecular networks, consistency scores were calculated, where a high consistency score indicated accurate results for the regulatory effects analysis. For upstream regulators, the P-value of overlap < 0.05 was set as the threshold. The algorithm for calculating the Z-scores and P-values of overlap were used from a previous study [22]. Functional analyses were performed using IPA between AEVs derived from OGD astrocytes and AEVs derived from OGD astrocytes treated with P2Y_1_R-ANT and MCM.

### Image Acquisition and Quantification

Immunohistochemical analyses of rat coronal brain sections and immunocytochemical analyses of cultured microglia were performed using a Zeiss LSM 780 confocal laser-scanning microscope (Carl Zeiss, Jena, Germany). Three randomly selected locations within the peri-infarct area in the VI layer of the cerebral cortex per section were acquired under a 40× objective. The peri-infarct area was determined as the demarcated region 300 μm away from the ischemic core of each Hematoxylin–Eosin immunohistochemical stain. In the sham-operated rats, the regions corresponding to those of the MCAO rats (that were randomly selected) were analyzed. Images of primary microglia cultured on cover glasses were acquired using a 40× objective lens. The density of immunoreactive cells and areas were analyzed using Imaris software (Birplane, Zurich, Switzerland). To automatically generate colocalization data in IMARIS software, we applied a threshold cutoff that removed background level for each immunostained image. Manual thresholds of GFAP, Iba-1, MAP2, and pNFH were 1500, 1500, 5220, and 2540, respectively. In measuring the area of the positive region in each immunostained image, we applied a threshold cutoff numbers of voxels as 1 above 30, 1 above 50, 1 above 35, and 1 above 17, for GFAP, Iba-1, MAP2, and pNFH, respectively, by eliminating objects smaller than a predefined number of voxels.

### Statistics and Reproducibility

The values presented in this study are expressed as the mean ± standard deviation (SD). An unpaired *t*-test and one-way analysis of variance with post-hoc Tukey’s correction were repeatedly used to determine the significant differences between the two groups and among more than three groups, respectively. Survival curves were constructed using the Kaplan–Meier survival analysis. Differences in the survival curves were analyzed using the log-rank test. In the microarray analysis, statistical significance was repeatedly tested using an unpaired t-test with GeneSpring software. A P-value of < 0.05 indicated a statistically significant difference. Analyses were performed using the JMP software (version 11.2, SAS Institute Inc., Cary, NC, USA). Bar and line graphs were constructed using GraphPad Prism (version 5.0; GraphPad Software, Inc., La Jolla, CA, USA). We calculated the sample size based on α = 0.05 and β = 0.8 to obtain group sizes appropriate for detecting effect sizes in the range of 40–50% for cell culture models and 30–50% for in vivo models. Rats for immunohistochemical analysis in each treatment group were randomly selected from the rats that underwent physiological analyses. All experiments and measurements, including behavioral analyses, immunohistochemistry, immunocytochemistry, western blotting, and microarray data, were repeatedly carried out in a blinded and randomized manner.

### Data Availability

The datasets generated during and/or analyzed during the current study are available from the corresponding author on reasonable request.

## Results

### Temporal Profiles of Activated Microglia and Reactive Astrocytes in the peri-infarct area After MCAO

**Fig. 1:**
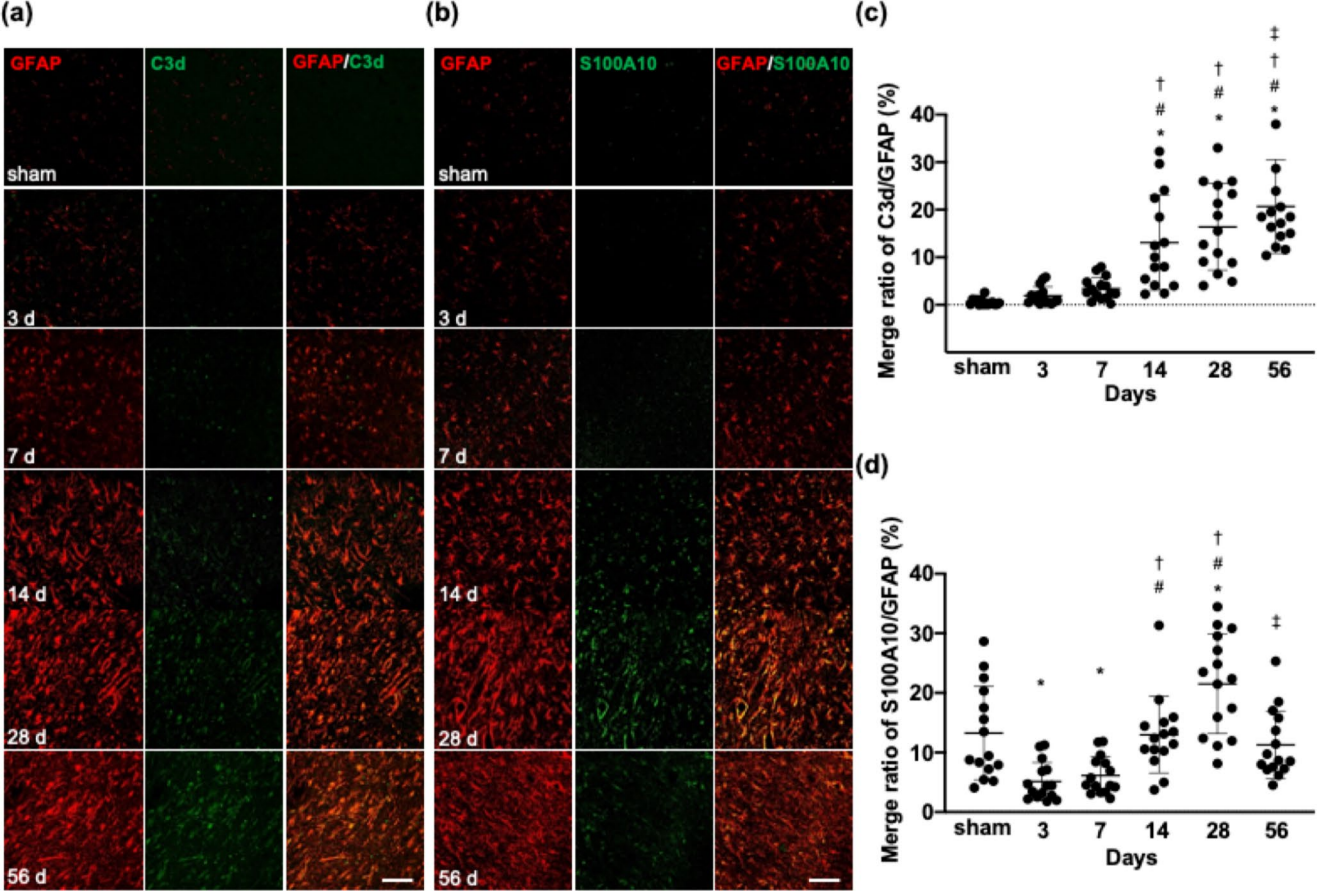
Temporal profile of reactive astrocytes with co-localization of C3d/S100A10 in the peri-infarct area. **(a), (b).** Double immunofluorescent confocal images of sham and the peri-infarct area at 3, 7, 14, 28 and 56 days after MCAO showing GFAP^+^ area (red), and C3d^+^ area (green, **a**) and S100A10^+^ area (green, **b**). Scale bar = 100 μm. **(c), (d).** Merge ratio of C3d /GFAP (**c**) and S100A10/GFAP (**d**). N = 5/group (three sections per rat, and total of 15 samples in each day after MCAO). Values are the mean ± SD. *P < 0.05 vs. sham, ^#^*P* < 0.05 vs. day 3, ^†^*P* < 0.05 vs. day 7, ^‡^*P* < 0.05 vs. day 28. MCAO = middle cerebral artery occlusion, GFAP = glial fibrillary acidic protein

Microglia and astrocytes are activated after stroke, and reactive astrocytes formatted glial scars in the chronic stage of infarction [4, 5, 23]. To explore whether microglia regulate astrocytes after ischemia, we examined the temporal profiles of microglial activation and reactive astrocytes, particularly in C3d and S100A10 expression, from the acute to chronic stages of stroke in rats subjected to MCAO. In layer IV of the peri-infarct area (Supplementary Fig. S1), Iba-1^+^ area increased and reached a peak 7 days after MCAO (12,320 ± 1635 µm^2^) and decreased in the chronic phase (Supplementary Fig. S2a, b). GFAP^+^ area increased from days 3 (5318 ± 1510 µm^2^) to 56 after MCAO (24,200 ± 2651 µm^2^, Supplementary Fig. S2a, c). The area of C3d^+^GFAP^+^ astrocytes increased from days 3 (2.1 ± 2.0% in GFAP^+^ astrocytes) to 56 (20.6 ± 10.7%) after MCAO, whereas the area of S100A10^+^GFAP^+^ astrocytes mildly decreased in the acute phase, increased on day 28 (22.8 ± 9.9%), and dropped 56 days (11.9 ± 6.0%) after MCAO (Fig. 1a–d). These data indicate that microglia and astrocytes increased during the acute and chronic stages of stroke, respectively. Reactive astrocytes with C3d and S100A10 together increased during the subacute and chronic infarction. In the chronic infarction at 56 days, reactive astrocytes with C3d expression further increased, whereas reactive astrocytes with S100A10 expression reduced by half of those with C3d.

### Microglial Activation Morphologies and Phenotypes After OGD and Treatment with P2Y_1_ Receptor Antagonists

Inhibition of P2Y_1_R by cytokines from activated microglia in astrocytes yields neuroprotection after traumatic brain injuries, whereas stimulation of microglial P2Y_1_ receptors exerts neuroprotection after ischemia [11, 24]. The immunocytochemistry analysis showed that the morphological profile of microglia was classified into three types: the resting phenotype for inactive microglia and bipolar/rod-shaped, and amoeboid phenotypes for activated microglia (Supplementary Fig. S3a) [25]. OGD and treatment with P2Y_1_R-ANT did not alter the expression of caspase-3, an apoptotic marker (Supplementary Fig. S4a). Resting microglia decreased 24 and 96 h after OGD, amoeboid microglia increased 24 h after OGD and decreased at 96 h after OGD, and bipolar/rod-shaped microglia increased at 96 h after OGD. These morphological changes were not dependent on treatment with P2Y_1_R-ANT (Supplementary Fig. S3b–d). The number of iNOS^+^ Iba-1^+^ cells increased 24 h after OGD, which was suppressed by treatment with P2Y_1_R-ANT. The number of arginase-1^+^ Iba-1^+^ cells decreased at 96 h after OGD compared to that seen with ischemic microglia treated with P2Y_1_R-ANT at 24 and 96 h (Supplementary Fig. S5a, b). These data indicate the drastic alterations in the morphologies of the resting to amoeboid microglial phenotypes at 24 h and the alterations in the morphologies to bipolar/rod-shaped microglia at 96 h after OGD. P2Y_1_R-ANT exerted modest effects on suppressing iNOS accumulation at 24 h and sustaining depleted arginase-1 at 96 h after OGD.

### Expression of C3d/S100A10 and Suppression Of Inflammatory Genes After OGD and Treatment with P2Y_1_ R-ANT and MCM in Reactive Astrocytes

**Fig. 2:**
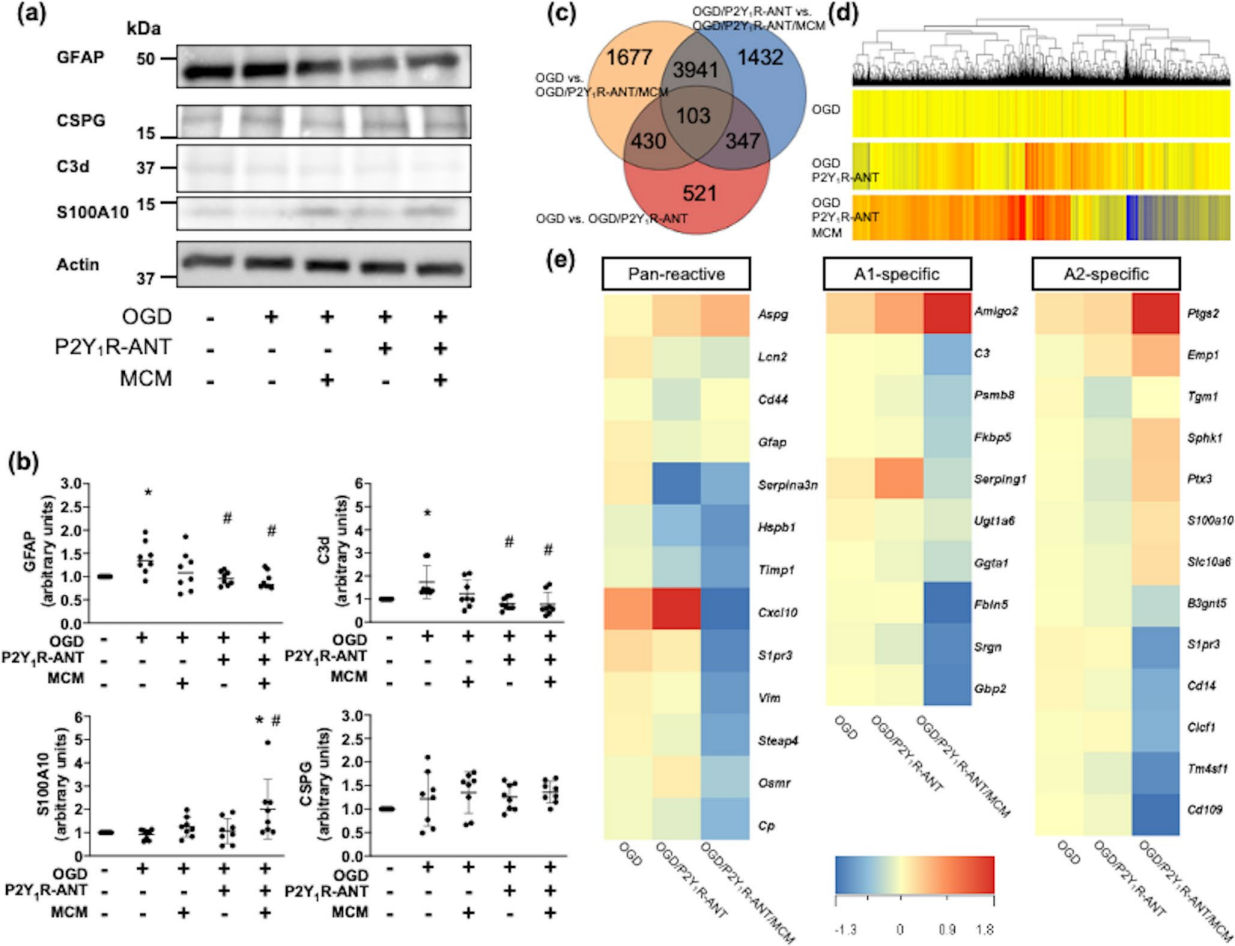
Changes of C3d/S100A10 levels, and pan-reactive and A1/A2 specific gene expression in reactive astrocytes after treatment with microglia-conditioned media (MCM) and P2Y_1_R-ANT. **(a), (b).** Representative images **(a)** and quantitative data **(b)** of western blots showing protein levels of GFAP, C3d, S100A10, and CSPG in non-OGD astrocytes, OGD astrocytes, OGD astrocytes treated with MCM, OGD astrocytes treated with P2Y_1_R-ANT, and OGD astrocytes treated with P2Y_1_R-ANT and MCM. β-actin was used as an internal control. ^*^P < 0.05 vs. non-OGD astrocyte, ^#^*P* < 0.05 vs. OGD astrocytes. **(c).** Venn diagram of mRNAs with upregulated (fold change ≥ 1.5) and downregulated (fold change ≤ 0.67) expressions in OGD astrocytes, OGD astrocytes treated with P2Y_1_R-ANT (1 mM), and OGD astrocytes treated with P2Y_1_R-ANT (1 mM) and MCM. **(d).** Heatmap of the entire mRNA expression in OGD astrocytes, OGD astrocytes treated with P2Y_1_R-ANT (1 mM), and OGD astrocytes treated with P2Y_1_R-ANT (1 mM) and MCM. **(e).** Heatmaps comparing the mean expression of pan-reactive, A1-specific, and A2 specific genes in OGD astrocytes; OGD astrocytes treated with P2Y_1_R-ANT (1 mM); and OGD astrocytes treated with P2Y_1_R-ANT (1 mM) and MCM. N = 4/group. Values are the mean ± SD. GFAP = glial fibrillary acidic protein, CSPG = chondroitin sulfate proteoglycans, OGD = oxygen–glucose deprivation, P2Y_1_R-ANT = P2Y_1_ receptor antagonist, MCM = microglial conditioned medium

We examined the changes in the expression of C3d and S100A10 in cultured astrocytes among non-OGD, OGD, OGD and treatment with MCM, OGD and treatment with P2Y_1_R-ANT, and OGD and treatment with P2Y_1_R-ANT and MCM groups. There were no significant differences in cell viability (Supplementary Fig. S4b). The protein levels of P2Y_1_R were not different after treatment with P2Y_1_R-ANT or MCM in cultured astrocytes (Supplementary Fig. S6a), while P2Y_1_R^+^GFAP^+^ astrocytes increased at 3 days and decreased thereafter 7 days in rat MCAO model (Supplementary Fig. S7a). OGD increased the level of GFAP (1.39 ± 0.34 [arbitrary units]), which was decreased by P2Y_1_R-ANT (0.96 ± 0.15 [arbitrary units]) and combined P2Y_1_R-ANT and MCM (0.92 ± 0.18 [arbitrary units], Fig. 2a, b). The protein levels of C3d were also increased in the OGD astrocytes (1.55 ± 0.54 [arbitrary units]) and were decreased by P2Y_1_R-ANT (0.79 ± 0.26 [arbitrary units]) and combined P2Y_1_R-ANT and MCM (0.77 ± 0.51 [arbitrary units], Fig. 2a, b). The level of S100A10 was increased in OGD astrocytes that were treated with P2Y_1_R-ANT and MCM (2.01 ± 1.27 [arbitrary units], Fig. 2a, b). Sole treatment of MCM did not alter the levels of GFAP, C3d, and S100A10. CSPG levels did not differ among the four groups (Fig. 2a, b). Thus, treatment with only P2Y_1_R-ANT decreased C3d levels, whereas a combination of P2Y_1_R-ANT and MCM reduced C3d levels and increased S100A10 levels in cultured astrocytes after OGD. TNF-α and IL-1β levels in astrocyte-conditioned medium assessed using ELISA were not different among the five groups (Supplementary Fig. S8a, b).

**Fig. 3:**
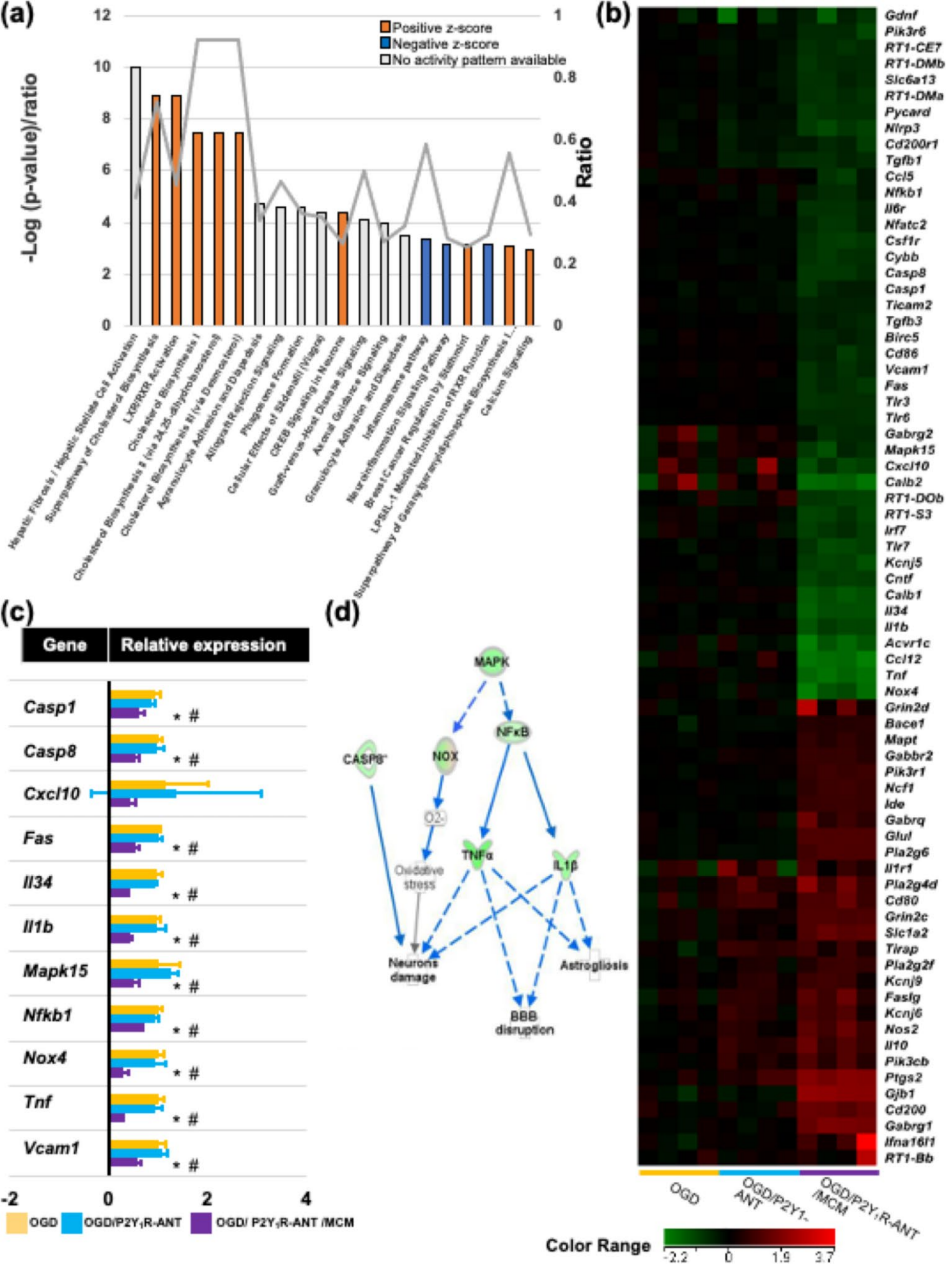
Change in inflammatory gene expression and pathway analysis. **(a).** Top 20 significant canonical pathways of the core analysis in IPA of most highly expressed genes in OGD astrocytes treated with P2Y_1_R-ANT (1 mM) and MCM relative to OGD astrocytes. Blue bars: negative z-score; orange bars: positive z-score; gray bars: no activity pattern available; white bars: activity of zero. **(b), (c).** Heatmap of mRNA-related Neuroinflammation Signaling expression, and quantitative analysis of representative mRNA-related Neuroinflammation Signaling in OGD astrocytes, OGD astrocytes treated with P2Y_1_R-ANT (1 mM), and OGD astrocytes treated with P2Y_1_R-ANT (1 mM) and MCM. ^*^P < 0.05 vs. OGD astrocyte, ^#^*P* < 0.05 vs. OGD astrocytes treated with P2Y_1_R-ANT. **(d).** Signaling pathway predicted by analyzing changes using IPA software in mRNA expression in the OGD astrocytes treated with P2Y_1_R-ANT and MCM relative to OGD astrocytes, or OGD astrocytes treated with P2Y_1_R-ANT. The functional networks were generated via IPA (QIAGEN Inc., https://www.qiagenbio-informatics.com/products/ingenuity-pathway-analysis). N = 4/group. Values are the mean ± SD. IPA = Ingenuity Pathway Analysis, P2Y_1_R-ANT = P2Y_1_ receptor antagonist, MCM = microglial conditioned medium, MAPK = mitogen-activated protein kinase, NF-κB = nuclear factor-κβ, TNF-α = tumor necrosis factor, IL-1β = interleukin-1β, NOX = nitrogen oxides, CASP8 = Caspase 8

To further explore the molecular mechanisms in ischemic astrocytes regulated by microglia or an inhibition of P2Y_1_R-ANT, a transcriptomic analysis was conducted to compare differentially expressed genes in OGD astrocytes treated with P2Y_1_R-ANT, and OGD astrocytes treated with a combination of P2Y_1_R-ANT and MCM relative to OGD astrocytes. In astrocytic mRNAs with marked differences among the three groups are shown in Fig. 2c. According to IPA [22], in the hierarchical clustering of mRNA expression among the three groups, a markedly altered abundance of mRNA was observed in OGD astrocytes with a combined treatment of P2Y_1_R-ANT and MCM relative to OGD astrocytes (Fig. 2d). Regarding the A1- and A2-phenotypic genes as well as pan-reactive genes in astrocytic populations [26, 27], our data indicated that ischemic astrocytes treated with P2Y_1_R-ANT and MCM downregulated the expressions of A1-specific genes, such as *psmb8*, *c3d*, *srgn*, *gbp2*, *fkbp5*, and *fbln5*; however, alterations in A2-specific genes were involved in the upregulation of *ptgs2* expression and downregulation of the expressions of *clcf1*, *cd14*, *emp1*, and *cd109* (Fig. 2e). In pan-reactive genes, *hspb1*, *timp1*, *cxcl10*, *s1pr3*, *vim*, *steap4*, and cp. expressions were downregulated in ischemic astrocytes treated with P2Y_1_R-ANT and MCM relative to ischemic astrocytes, whereas *serpina3n* expression was downregulated in ischemic astrocytes treated with P2Y_1_R-ANT (Fig. 2e). Canonical pathways included the ‘Neuroinflammation Signaling Pathway’ as the 16th most relevant and second downregulated pathway in OGD astrocytes treated with P2Y_1_R-ANT and MCM relative to OGD astrocytes and the ninth most relevant and first downregulated pathway in OGD astrocytes treated with P2Y_1_R-ANT and MCM, relative to OGD astrocytes and OGD astrocytes treated with P2Y_1_R-ANT (Fig. 3a, Supplementary Fig. S9a). The Neuroinflammation Signaling Pathway was not involved in 19 canonical pathways in OGD astrocytes treated with P2Y_1_R-ANT relative to OGD astrocytes (Supplementary Fig. S9b). We found that 74 of the known inflammatory molecules were substantially altered in OGD astrocytes treated with P2Y_1_R-ANT and MCM, relative to OGD astrocytes. The heat map and relative expression of inflammatory molecules among the three groups are shown in Fig. 3b and c. Finally, a functional network implicated the downregulation of the mitogen-activated protein kinase (MAPK)/ NF-κB/tumor necrosis factor (TNF)-α, or MAPK/NF-κB/interleukin (IL)-1β pathways, which inhibited blood–brain barrier disruption, astrogliosis, and neuronal damage (Fig. 3d).

### Intracerebral Administration of the P2Y_1_R Antagonist Alone does not Improve Stroke Recovery

To examine the effects of P2Y_1_R-ANT or the combination of P2Y_1_R-ANT and MCM on glial scars in the peri-infarct area and motor function during the chronic stage of stroke, we examined the effects of treatment with P2Y_1_R-ANT alone. Using an osmotic minipump (Alzet Corporation, Cupertino, CA), we administered P2Y_1_R-ANT into the peri-infarct area of MCAO rat brains for 14 days (0.25 ml/h) from 7 to 21 days after MCAO, which is when microglial activation reaches a peak, as well as when astrocytic activation and axonal outgrowth are started, according to the current data (Supplementary Fig. S2a, b, S10a) and our previous work [12]. We found no significant differences in the survival rates, body weights, and the modified mNSSs between vehicle-treated and 1 and 10 nM P2Y_1_R-ANT–treated rats subjected to MCAO (Supplementary Fig. S10b–d). Double immunohistochemical staining showed that the area of GFAP^+^C3d^+^ astrocytes in the peri-infarct area were significantly decreased after treatment with 1 (2.89 ± 1.57%) and 10 mM (2.93 ± 1.08%) of P2Y_1_R-ANT compared to that in the vehicle-treated group, whereas the area of GFAP^+^S100A10^+^ astrocyte were not significantly different after treatment with P2Y_1_R-ANT among the three groups (Supplementary Fig. S10e, f). These data suggest that treatment with P2Y_1_R-ANT alone reduced reactive astrocytes with the C3d but not S100A10 markers and did not improve motor recovery from stroke.

### EVs Derived from Reactive Astrocytes with anti-inflammatory Properties Regulate Glial Scars

**Fig. 4:**
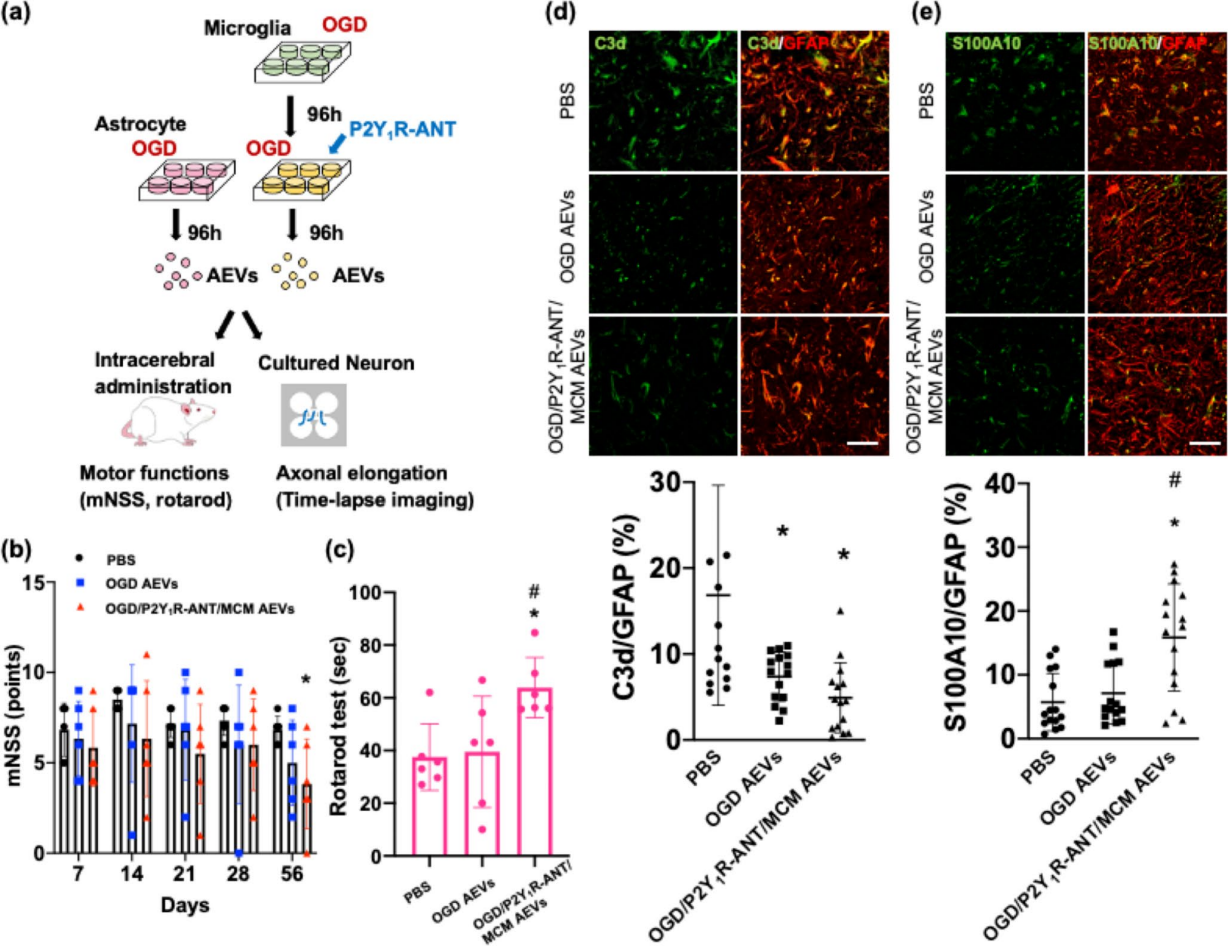
Significance of AEVs derived from anti-inflammatory astrocytes to the peri-infarct area after MCAO. **(a).** The experimental scheme of isolating AEVs from OGD astrocytes treated with MCM and P2Y_1_R-ANT and their application for rats subjected to MCAO and cultured cortical neurons. **(b), (c).** Modified neurological severity score (**b**) and latency to fall off the rotarod at 56 days after MCAO (**c**) in PBS treatment, treatment with AEVs derived from OGD astrocytes (100 µg), and OGD astrocytes treated with MCM and P2Y_1_R-ANT (100 µg) in rats subjected to MCAO. N = 6–7/group. Values are the mean ± SD. **(d), (e).** Double immunofluorescent confocal images and quantitative data of the peri-infarct area at 56 days after MCAO with intracerebral administration of PBS, 100 µg AEVs derived from OGD astrocytes, and 100 µg AEVs derived from OGD astrocytes treated with MCM and P2Y_1_R-ANT, showing C3d^+^ area (green) (**d**) and S100A10^+^ area (green) (**e**), and co-localized with GFAP^+^ area (yellow). Merge ratio of C3d /GFAP (**d**), and S100A10/GFAP (**e**). N = 5/group (three sections per rat, and total of 15 samples in each group). Values are the mean ± SD. ^*^P < 0.05 vs. PBS-treated rats, ^#^*P* < 0.05 vs. rats treated with AEVs derived from OGD astrocytes. Scale bar = 100 μm AEVs = astrocytic extracellular vesicles, MCAO = middle cerebral artery occlusion, P2Y_1_R-ANT = P2Y_1_ receptor antagonist, MCM = microglia-conditioned medium, GFAP = glial fibrillary acidic protein, OGD = oxygen–glucose deprivation

To analyze the therapeutic effects of AEVs derived from ischemic astrocytes, we next stereotaxically administered AEVs from cultured OGD astrocytes and OGD astrocytes treated with P2Y_1_R-ANT and MCM according to the previous study [28], because modification of gene expression, especially in the downregulation of the expressions of inflammatory-related genes, was identified in OGD astrocytes treated with P2Y_1_R-ANT and MCM, relative to OGD astrocytes. Using PBS as a control, we examined neurological deficits and immunohistochemical changes in rats subjected to MCAO after stereotaxic treatment with AEVs (Fig. 4a). Tetraspanin proteins and miRNA cargo in these AEVs were analyzed.

Among the rats subjected to MCAO and stereotaxically treated with PBS, AEVs derived from cultured astrocytes challenged to OGD and those derived from astrocytes with OGD and treatment with P2Y_1_R-ANT and MCM showed no significant differences in survival rates and body weight (Supplementary Fig. S11a, b). The mNSSs showed that AEVs derived from OGD astrocytes treated with P2Y_1_R-ANT and MCM displayed significant improvements 56 days after MCAO in neurological deficits, compared to that in the PBS-treated group (Fig. 4b). MCAO-subjected rats treated with AEVs produced from OGD astrocytes with a treatment of P2Y_1_R-ANT and MCM exhibited a longer latency to fall off the rotarod than MCAO-subjected rats treated with PBS (Fig. 4c). The infarct volumes did not differ among the three treatment groups (Supplementary Fig. S12a, b). Double immunohistochemistry in the peri-infarct area 56 days after MCAO showed that AEVs derived from OGD astrocytes treated with P2Y_1_R-ANT and MCM significantly reduced C3d^+^GFAP^+^ astrocytes (4.91 ± 4.07% vs. 16.84 ± 12.79%) and increased S100A10^+^ astrocytes compared to that in the PBS-treated group (15.85 ± 8.41% vs. 5.72 ± 4.51%) and that seen in the AEVs derived from OGD astrocytes (7.11 ± 4.82%) (Fig. 4d, e). AEVs derived from OGD astrocytes also decreased C3d^+^GFAP^+^ astrocytes (7.37 ± 2.86%) compared to those in the PBS-treated group (Fig. 4d, e). Thus, treatment with AEVs derived from cultured ischemic astrocytes with anti-inflammatory properties and the reduction of C3d and increase of S100A10 further downregulated C3d and increased S100A10 marker expression in reactive astrocytes in the peri-infarct area of chronic phase rats subjected to MCAO, which thereby facilitated stroke recovery. Conversely, AEV derived from OGD astrocytes reduced C3d expression in reactive astrocytes but did not improve functional recovery after stroke.

**Fig. 5:**
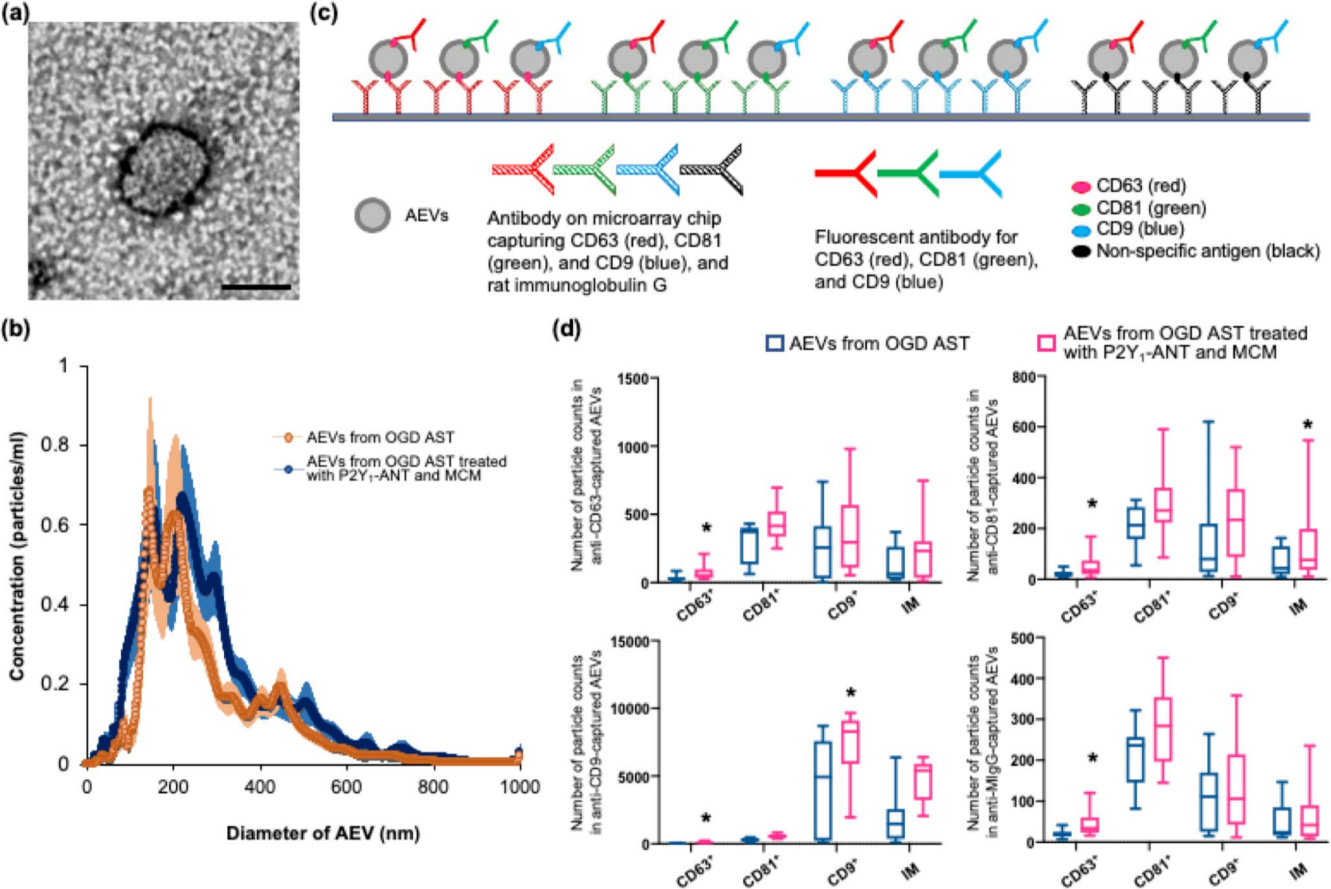
Profile of AEVs derived from cultured astrocytes. **(a).** Representative image of AEVs on transmission electron microscopy isolated from astrocyte culture media. Scale bar = 100 nm. **(b).** Particle size distribution of AEVs using NanoSight particle tracking analysis. **(c).** Schematic illustration, showing AEV samples captured on the surface of chips equipped with anti-CD63 (diagonal red line), CD 81 (diagonal green line), CD9 (diagonal blue line), and MIgG, combined with antibody-based microchip capturing CD63 (solid red line), CD81 (solid green line), and CD9 (solid blue line) with fluorescence detection. **(d).** Quantitative data of the number of particle counts in the presence of CD9, CD63, and CD81 on AEV subpopulations captured using antibody spots against CD63, CD81, CD9, or MIgG on the microarray. N = 3/group. Values are the mean ± SD. *P < 0.05 vs. AEVs derived from OGD astrocytes. AEV = astrocytic extracellular vesicles, OGD = oxygen–glucose deprivation, IM = immunoglobulin, as a non-specific binding control

Transmission electron microscopy images showed that encapsulated particles were found in cells harvested from cultured astrocytes after OGD (Fig. 5a). Figure 5b showed that small-to-medium-sized particles were substantially abundant in AEVs from OGD astrocytes and those treated with P2Y_1_R-ANT and MCM. Transmembrane tetraspanin proteins, including CD9, CD63, and CD81, are a major class of EV-expressing molecules [29, 30]. In the ExoView chip-based analysis, AEVs were incubated on a microarray equipped with antibody spots against CD63, CD81, and CD9, or mouse immunoglobulin G (MIgG, a non-specific binding control), combined with an antibody-based microchip capturing CD63, CD81, and CD9 with fluorescence detection (Fig. 5c). The majority of AEVs were captured by anti-CD63, CD 81, and CD9 antibody-coated spots, and by the MIgG-coated spot with fluorescent detection with CD63, CD 81, and CD9, indicating that the majority of AEVs expressed tetraspanin protein (Fig. 5d). The numbers of CD63^+^ immunofluorescent particles captured by anti-CD63, -CD81, -CD9, and -MIgG antibodies, CD9^+^ immunofluorescent particles captured by anti-CD9 antibodies, and IM captured by anti-CD81 antibodies were significantly higher in AEVs from OGD astrocytes treated with P2Y_1_R-ANT and MCM than in AEVs from OGD astrocytes (Fig. 5d). Among these, CD63 and CD9 expressions were suggested to be particularly related to AEVs from OGD astrocytes treated with P2Y_1_R-ANT and MCM, because of significant increases in CD63^+^CD63^+^ and CD9^+^CD9^+^ immunofluorescent particles.

**Fig. 6:**
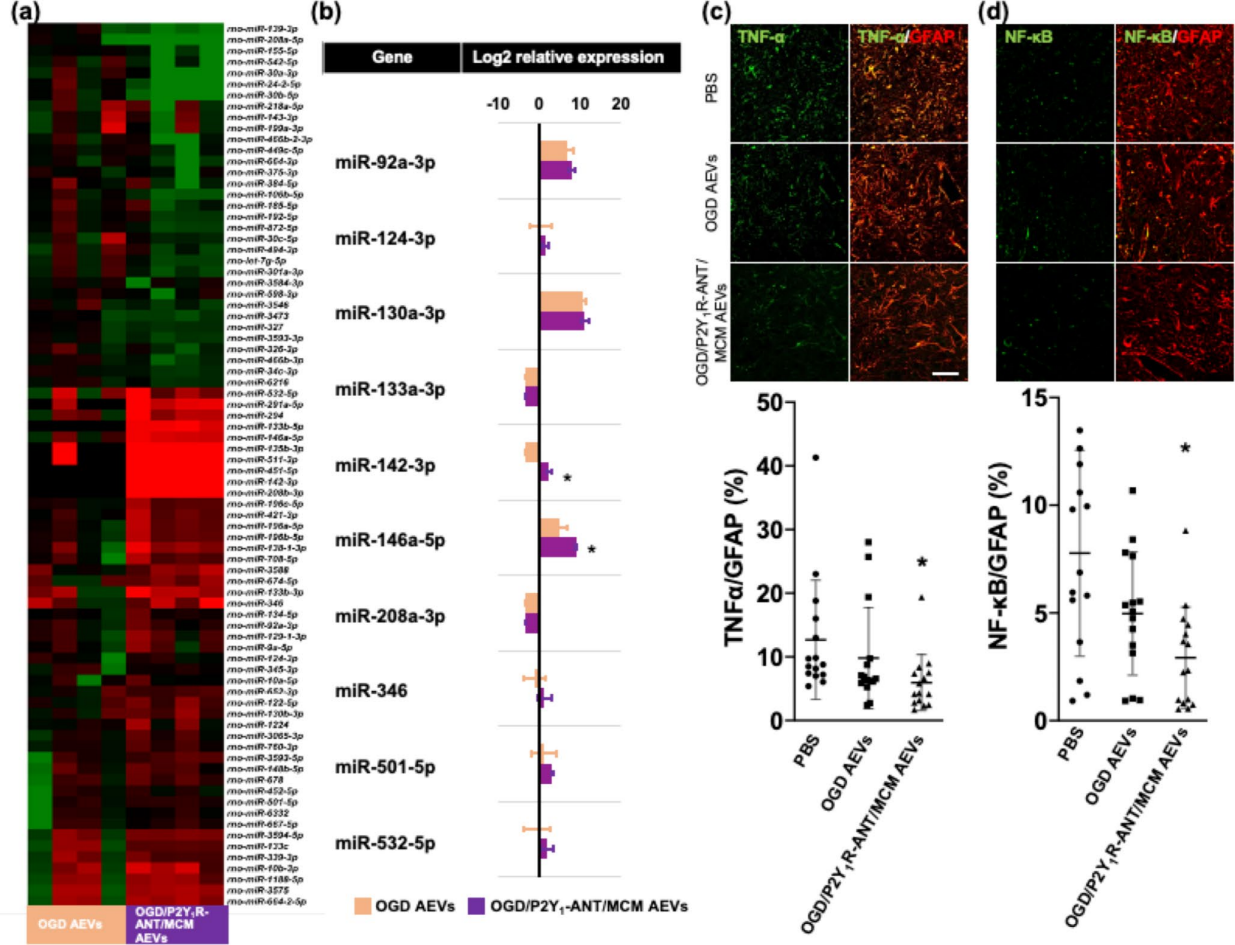
Expression of microRNAs (miRNAs) in AEVs and inflammatory regulation in peri-infarct glial scars. **(a).** Heatmap of miRNA profiles on AEVs derived from OGD astrocytes and OGD astrocytes treated with P2Y_1_R-ANT and MCM. **(b).** Quantitative analysis of representative miRNAs related to ‘Inflammatory Response’ in AEVs derived from OGD astrocytes treated with P2Y_1_R-ANT and MCM, relative to AEVs derived from OGD astrocytes. N = 4/group. Values are the mean ± SD. *P < 0.05 vs. AEVs derived from OGD astrocytes. **(c), (d).** Double immunofluorescent confocal images and quantitative data of the peri-infarct area 56 days after MCAO with intracerebral administration of PBS, 100 µg AEVs derived from OGD astrocytes, and 100 µg AEVs derived from OGD astrocytes treated with MCM and P2Y_1_R-ANT, showing TNFα^+^ area (green) (**c**) and NF-κB^+^ area (green) (**d**), and co-localized with GFAP^+^ area (yellow). N = 5/group (three sections per rat, and total of 15 samples in each group). Values are the mean ± SD. ^*^P < 0.05 vs. PBS-treated rats. Scale bar = 100 μm AEVs = astrocytic extracellular vesicles, P2Y_1_R-ANT = P2Y_1_ receptor antagonist

EVs transfer miRNAs in their cargo from parent to recipient cells [13, 31]. A miRNA microarray analysis showed that AEVs derived from OGD astrocytes treated with P2Y_1_R-ANT and MCM had 47 and 33 miRNAs with upregulated and downregulated expressions, respectively, relative to AEVs from OGD astrocytes (Fig. 6a, b). Considering the Top Diseases and Bio Functions in the IPA, the inflammatory response included 11 categories and 11 miRNAs (Supplementary Table S1). Among these miRNAs, miR-146a-5p displayed substantial expression and higher fold changes in AEVs derived from OGD astrocytes treated with P2Y_1_R-ANT and MCM relative to AEVs from OGD astrocytes (Fig. 6b). miR-146a-5p inhibits the NF-κB signaling pathway, whereas NF-κB activation stimulates the production and release of TNF-α [32, 33]. We found that AEVs derived from OGD astrocytes treated with P2Y_1_R-ANT and MCM exhibited a siginificant reduction in TNF-α^+^GFAP^+^ astrocytes (6.85 ± 2.63%) and NF-κB^+^GFAP^+^ (3.06 ± 1.95%) at 56 days after MCAO (Fig. 6c, d). Collectively, a combination of P2Y_1_R-ANT and MCM induced reactive astrocytes with anti-inflammatory properties, which released AEVs with unique profiles of CD63 and CD9 tetraspanins, and miR-146a-5p. In particular, miR-146a-5p inhibited NF-κB signaling and the production and release of TNF-α.

### Axonal outgrowth after treatment with AEVs from reactive astrocytes with anti-inflammatory properties in vivo and in vitro

**Fig. 7:**
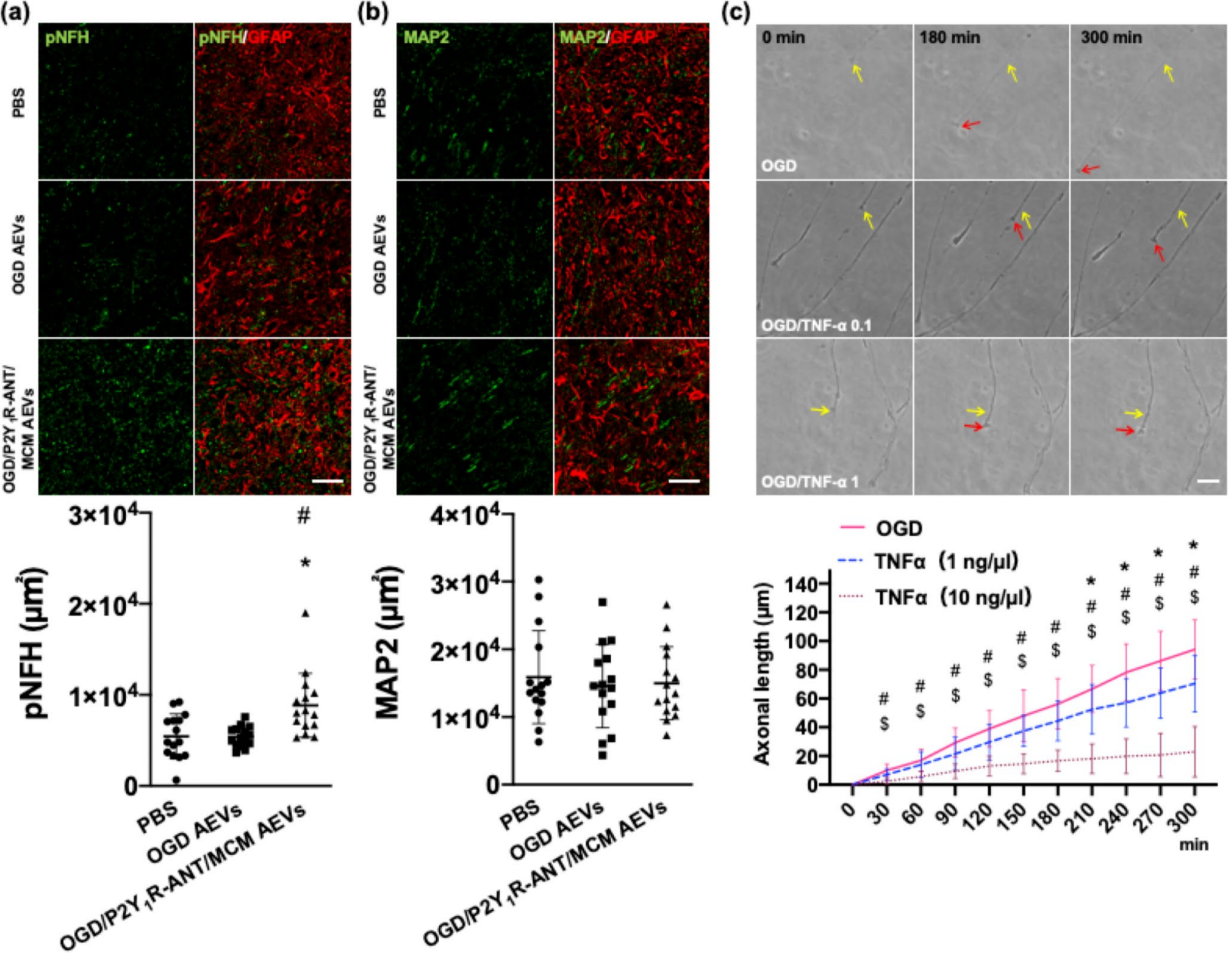
Axonal outgrowth after AEV treatment and hindering by TNF-α **(a), (b).** Double immunofluorescent confocal images and quantitative data of the peri-infarct area 56 days after MCAO with intracerebral administration of PBS, 100 µg AEVs derived from OGD astrocytes, and 100 µg AEVs derived from OGD astrocytes treated with MCM and P2Y_1_R-ANT, showing pNFH^+^ axons (green) (**a**) and MAP2 cells^+^ (green) (**b**), with GFAP. N = 5/group (three sections per rat, and total of 15 samples in each group). Values are the mean ± SD. Scale bar = 100 μm. **(c).** Representative time-lapse microscopic images and quantitative data of primary cortical neurons in a microfluidic chamber showing axonal elongation (distance from yellow arrow to red arrow) in OGD neurons, OGD neurons treated with 1 ng/µl of TNF-α, and OGD neurons treated with 10 ng/µl of TNF-α. N = 3/group. Values are the mean ± SD. Scale bar = 20 μm. Quantitative data of axonal elongation per 30 min prior to 96 h after OGD. ^*^P < 0.05, OGD neurons treated with 1 ng/µL of TNF-α vs. OGD neurons; ^#^*P* < 0.05, OGD neurons treated with 10 ng/µL of TNF-α vs. OGD neurons; ^$^*P* < 0.05, OGD neurons treated with 10 ng/µL of TNF-α vs. OGD neurons treated with 1 ng/µL of TNF-α. AEVs = astrocytic extracellular vesicles, MCAO = middle cerebral artery occlusion, OGD = oxygen–glucose deprivation, P2Y_1_R-ANT = P2Y_1_ receptor antagonist, MCM = microglia-conditioned medium, GFAP = glial fibrillary acidic protein, pNFH = phosphorylated neurofilament heavy chain

**Fig. 8:**
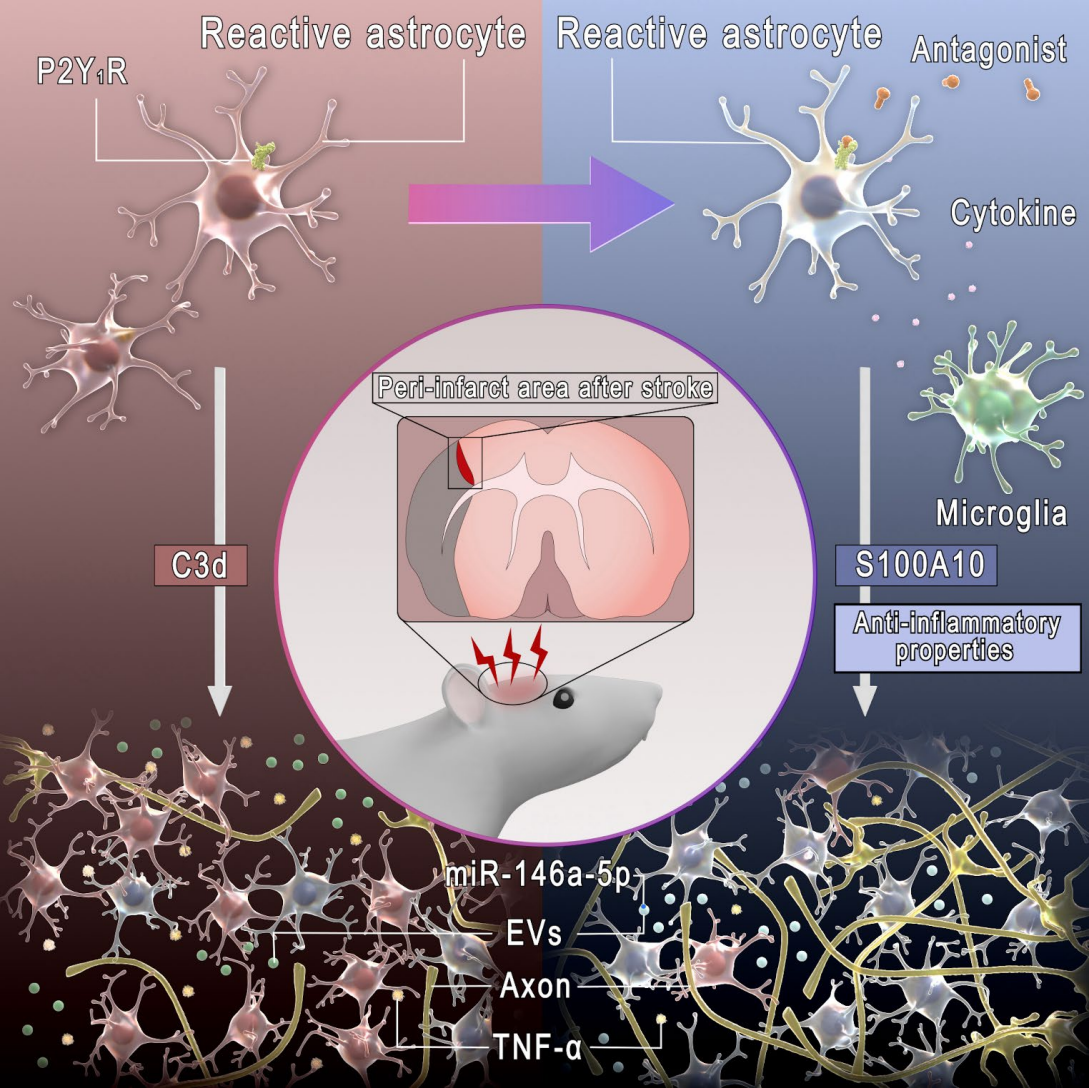
Diagram depicting the findings of the present study. Microglia and inhibition of P2Y_1_R regulate reactive astrocytes by transforming C3d/S100A10 expression and suppressing neuroinflammation. AEVs derived from reactive astrocytes with anti-inflammatory properties possessing miR-146a-5p regulate glial scars by suppressing NF-κB and TNF-α, which is permissive for axonal outgrowth and improves stroke recovery

In the peri-infarct area of rat MCAO model, pNFH^+^ axons were substantially increased after the administration of AEVs derived from OGD astrocytes treated with P2Y_1_R-ANT and MCM (10794.0 ± 3528.3 µm^2^) compared to those in MCAO rats treated with AEVs derived from OGD astrocytes (5273.1 ± 1015.1 µm^2^) and those in PBS-treated MCAO rats (5981.5 ± 1989.5 µm^2^), whereas the expression of microtubule-associated protein 2^+^ was not noted (Fig. 7a, b). We cultured cortical neurons in a microfluidic chamber. After OGD for 3 h, we placed AEVs derived from OGD astrocytes and those derived from OGD astrocytes treated with P2Y_1_R-ANT and MCM into the somal (3 × 10^9^) and axonal compartments (3 × 10^8^). After following our previous protocol [12], our time-lapse data showed that axonal elongation every 30 min and the total elongation length at 300 min in OGD neurons, OGD neurons treated with OGD astrocyte-derived AEVs, and OGD neurons treated with AEVs from OGD astrocytes treated with P2Y_1_R-ANT and MCM did not exhibit significant differences (7.89 ± 3.90 μm vs. 9.16 ± 3.81 μm vs. 9.81 ± 3.10 μm, and 78.88 ± 38.95 μm vs. 91.64 ± 38.06 μm vs. 98.09 ± 31.00 μm, respectively) (Supplementary Fig. S13a, b). These results suggest that axonal outgrowth after ischemia is not a direct effect of AEVs but is a secondary impact of the anti-inflammatory regulation of glial scars treated with AEVs. To test this hypothesis, we used TNF-α in cultured cortical neurons following OGD. The pNFH protein levels significantly decreased in cultured cortical neurons with TNF-α treatment in a dose-dependent manner, whereas the NF-κB levels were not different (Supplementary Fig. S14a-c). To further analyze whether TNF-α hindered axonal outgrowth after ischemia, 1 and 10 ng/µl of TNF-α were applied in axonal compartments after OGD. Axonal elongation every 30 min and the total elongation length at 300 min were significantly suppressed in OGD neurons treated with 10 ng/µl of TNF-α (2.49 ± 1.99 μm and 22.97 ± 17.48 μm) compared to that in OGD neurons treated with 1 ng/µl of TNF-α (7.02 ± 3.97 μm and 70.38 ± 19.87 μm) and OGD neurons (9.98 ± 4.30 μm and 94.20 ± 20.47 μm) (Fig. 7c). Therefore, TNF-α hindered axons regardless of NF-κB signaling in cultured cortical neurons after ischemia. Collectively, our data indicated that AEVs derived from ischemic astrocytes with the downregulation of the MAPK/NF-κB pathway with the TNF-α or IL-1β pathways induced anti-inflammatory effects with augmented miR-146a-5p expression, which inhibited NF-κB signaling, suppressed the release of TNF-α from reactive astrocytes, modulated glial scars to be permissive for axonal outgrowth, and thereby improved motor recovery (Fig. 8).

## Discussion

In this study, we demonstrated that the inhibition of chronic neuroinflammation in peri-infarct glial scars could be permissive for axonal outgrowth and enhance functional recovery. Our data suggest that the reactive astrocytes combined with increased S100A10 levels, reduced C3d levels, and suppressed MAPK/NF-κB/TNF-α or IL-1β signaling pathways facilitated axonal outgrowth and functional recovery, whereas the suppression of C3d expression in reactive astrocytes alone was insufficient. Furthermore, the intracerebral administration of AEVs derived from ischemic astrocytes with anti-inflammatory properties, including CD63 and CD9 tetraspanins and miR-146a-5p, induced anti-inflammatory responses in the peri-infarct glial scars and facilitated stroke recovery.

The present data indicate that activated microglia increased in the peri-infarct area in the acute phase of stroke, preceding glial scar formation. In vitro, resting microglia decreased and amoeboid microglia at 24 h after OGD and bipolar/rod-shaped microglia at 96 h after OGD increased. Although P2Y_1_R-ANT did not transform the morphology of microglia after OGD, P2Y_1_R-ANT sustained baseline levels of the M1 marker at 24 h after OGD and the M2 marker at 96 h after OGD. Amoeboid and bipolar/rod-shaped microglia are related to activated microglia, which are found in the early stages of Parkinson’s disease, Alzheimer’s disease, and focal transient ischemia. [25] As for the M1/M2 phenotypes, in a rodent stroke model, M1 microglia increased in the first 14 days and then decreased, whereas M2 microglia increased on day 1, reached a peak at 5–7 days, and then decreased [34, 35]. P2Y_1_R is a G-protein coupled metabotropic receptor for adenosine 5’-triphosphate (ATP), which is known as the intracellular energy currency [36]. It has been shown that P2Y_1_R-ANT suppresses microglial activation but fails to improve functional recovery after traumatic brain injury, whereas the stimulation of P2Y_1_R on microglia suppresses TNF-α production and exerts neuroprotection in the early stage of stroke [24, 37, 38]. We elucidated that the inhibition of P2Y1R activity induced modest effects, including the early suppression of inflammatory marker and late sustained the level of restorative marker after ischemia, but did not transform the morphological alterations in microglia induced by ischemia. The association of microglial morphological profiles with the alteration of inflammatory/restorative molecules has yet to be elucidated, and further studies are warranted.

Microglia induce neuroprotection in astrocytes by producing cytokines that inhibit P2Y_1_R in astrocytes after traumatic brain injury [11]. In recent years, the prevailing idea that bipolarity of microglia (M1/M2 phenotype) and astrocytes (A1/A2 phenotype) was proposed [26]. Meanwhile, diversity of molecular expression in astrocytes has been considered more critical [7], along with latest progress in transcriptomics technologies [8, 9]. Early research of them showed that *Lcn2* and *Serpina3n* were induced in astrocytes at 1 day after MCAO [10]. Emerging data with single-cell RNA sequencing has revealed that five and seven subgroups were detected in healthy and early ischemic mouse brains, respectively [8, 9], and *Cyr61* might be related to cell death at 24 h after MCAO [9]. As per our transcriptomic data, P2Y_1_R-ANT alone suppressed the expressions of only one pan-reactive gene and none of the A1- and A2-specific genes that were defined in previous studies [26, 27], whereas a combination of P2Y_1_R-ANT and MCM suppressed the expressions of approximately one half of pan-reactive, A1-, and A2- specific genes. Importantly, some of these genes (including s*erpina3n*, *hspb1*, *gbp2*, *s1pr3*, and *clcf1*) were shown to exert neuroinflammation in astrocytes [39–42]. Thus, reactive astrocytes after ischemia in our experiment were not consistent with A1- and A2 phenotypes [26, 27] and indicated anti-inflammatory properties, especially in ischemic astrocytes treated with P2Y_1_R-ANT and MCM. Furthermore, pathway analysis indicated that a combination of P2Y_1_R-ANT and MCM suppressed inflammatory pathways, including the MAPK/NF-κB/TNF-α or IL-1β signaling in reactive astrocytes after ischemia. The association of synergic effect of P2Y1R-ANT and MCM with transformation of phenotype expressing C3d to S100A10 in reactive astrocytes is possibly related to anti-inflammation, but still unknown. TNF-α is a pro-inflammatory cytokine and the accumulation of NF-κB has deleterious effects on neurological diseases [43, 44]. On the contrary, it was shown that TNF-α increased NF-κB and improved neural viability after ischemia; NF-κB is implicated in neural development [45, 46]. Although TNF-α and NF-κB have pleiotropic roles, our data indicated that suppression of NF-κB together with these inflammatory pathways could be promising for stroke recovery.

Our data demonstrated that AEVs derived from ischemic astrocytes treated with P2Y_1_R-ANT and MCM contained more mir-146a, and CD63 and CD9 tetraspanins. EVs play a role in intercellular communication between glial cells and neurons, different types of glial cells, and the same types of glial cells [12, 13]. Intriguingly, EVs transfer their cargo, including miRNAs, and mediate signaling pathways in recipient cells. Specific molecules in EVs have been shown to exert angiogenesis, neurogenesis, axonal outgrowth, and anti-inflammatory effects [16]. Of these, the majority of EVs are derived from MSC. The overexpression of specific miRNAs, including the miR-17-92 cluster, miR-133b, miR-184, and miR-210 in cargo-regulated molecular signaling in ischemic brains, further enhances functional recovery after stroke [15, 16, 28, 47]. AEVs have also been shown to improve stroke recovery [12, 47, 48]. Emerging data have indicated that acute treatment with AEVs promote axonal regrowth, the reorganization in the somatosensory cortex, and functional recovery in the chronic phase of stroke, in which specific biomolecules were identified using proteomic analysis [48]. In our previous study, the inhibition of semaphorin 3 A suppressed the activation of astrocytes and downregulated miR-30c-2-3p and miR-326-5p expressions in astrocyte-derived exosomes, which enhanced axonal elongation in ischemic neurons by increasing prostaglandin D2 synthase activity [12]. Meanwhile, tetraspanins are known markers of EV populations and are related to protein sorting into EVs [49]. CD9 plays a role in tumor progression and metastasis [50]. The stimulation of the choroid plexus by amyloid-β oligomers increases the release of CD81^+^ and CD9^+^CD81^+^ EVs with pro-inflammatory proteins [51]. CD63 induces the biogenesis of EVs, whereas CD63 expression facilitates the release of EVs in Down syndrome [52, 53]. These data suggest that the expression of tetraspanins in EVs may have stronger biological implications. However, the link between tetraspanins and miRNA cargo in EVs remains to be elucidated.

Glial scars have been considered to critically hinder axonal outgrowth after CNS injury via physiological barriers and production of several inhibitory molecules [5, 54]. It has also been shown that the ablation of glial scars fails to promote motor recovery and that *cspg4* and *cspg5* are essential for axonal outgrowth after spinal cord injury. [6] We found that the peri-infarct glial scars in the chronic stage of stroke were primarily composed of reactive astrocytes with C3d markers when those with the expression of S100A10 decreased. AEVs derived from ischemic astrocytes treated with P2Y_1_R-ANT and MCM containing miR-146a exerted anti-inflammatory effects, reversed the expression of C3d/S100A10 in reactive astrocytes in the post-stroke glial scars, and thereby improved motor function, whereas P2Y_1_R-ANT solely suppressed C3d expression but did not increase S100A10 levels, which did not facilitate stroke recovery. Meanwhile, it was shown that AEVs derived from OGD astrocytes suppressed GFAP expression in astrocytes and increased neurite outgrowth after stroke [12, 47]. Our data showed that AEVs derived from OGD astrocytes reduced C3d expression, but not improved stroke recovery in the current study.

TNF-α triggers NF-κB signaling, and the activation of NF-κB signaling mediates TNF-α production [32, 33]; miR-146a represses NF-κB signaling [55]. We found that TNF-α was chemorepellent for axonal outgrowth after ischemia; the miR-146a in AEVs could inhibit NF-κB signaling and repress the inflammatory domino effect via TNF-α production in reactive astrocytes. Treatment with AEVs at 7 days after MCAO modulated the post-stroke glial scars as permissive for axonal outgrowth, which improved stroke recovery without reducing infarct size. The majority of concepts in previous studies regarded the reduction of infarct area by treating with EVs in the acute phase [13–16, 48]. EVs have a comparable treatment effect and safety compared to cell therapies for stroke [15, 16]. Given the clinical perspectives following acute therapy for stroke, AEV treatment in the subacute phase could be a candidate for facilitating functional recovery in stroke therapy.

Although our results are proof-of-concept study, there are several limitations. One limitation of our study was that the conditions of activated microglia and reactive astrocytes in the peri-infarct area after MCAO and those of cultured microglia and astrocytes were not consistent. P2Y_1_R-ANT was applied immediately after OGD in vitro, whereas P2Y_1_R-ANT was administered at 7 days in rats subjected to MCAO. MCM contained 10% fetal bovine serum (FBS), which is composed of a variety of fatty acids; this could affect molecular expression in cultured astrocytes administered with MCM, as well as cultured microglia. In fact, several pathways related to cholesterol biosynthesis were highly relevant in OGD astrocytes treated with P2Y_1_R-ANT and MCM, relative to OGD astrocytes, and OGD astrocytes treated with P2Y_1_R-ANT (Fig. 3a, Supplementary Fig. S8a). Another limitation was that the current study did not fully analyze the crosstalk between resident microglia and astrocytes in the peri-infarct area, especially in terms of the secondary effect of resident microglia for reactive astrocytes after MCAO, and treatment with P2Y_1_R-ANT or AEVs. Future studies with single-cell RNA sequencing for post-stroke peri-infarct glial scars in MCAO rats to analyze altered gene expression in different cell types and interaction between different are warranted.

In conclusion, an inhibition of P2Y_1_R alone modulated microglia and astrocytes by altering some reactive molecules after ischemia in vitro but did not improve stroke recovery in a rat MCAO model. Microglia, with an inhibition of P2Y_1_R, regulate reactive astrocytes and chronic glial scar formation by suppressing neuroinflammation. AEVs derived from reactive astrocytes with anti-inflammatory properties possess CD63 and CD9 tetraspanins and miR-146a-5p, which regulate glial scars by suppressing NF-κB and TNF-α, which permit axonal outgrowth, thereby improving stroke recovery. The current data suggest that AEVs delivering miR-146a-5p have potential application in stroke recovery.

### Electronic Supplementary Material

Below is the link to the electronic supplementary material.

Supplementary Material 1

## Data Availability

The datasets generated during and/or analyzed during the current study are available from the corresponding author on reasonable request.
