# Correction to: Astrocytic Extracellular Vesicles Regulated by Microglial Inflammatory Responses Improve Stroke Recovery

**DOI:** 10.1007/s12035-023-03657-5

**Published:** 2023-09-20

**Authors:** Chikage Kijima, Toshiki Inaba, Kenichiro Hira, Nobukazu Miyamoto, Kazuo Yamashiro, Takao Urabe, Nobutaka Hattori, Yuji Ueno

**Affiliations:** 1 Department of Neurology, Juntendo University Faculty of Medicine, Tokyo, Japan; 2 Department of Neurology, Juntendo University Urayasu Hospital, Chiba, Japan; 3 Neurodegenerative Disorders Collaborative Laboratory, RIKEN Center for Brain Science, Saitama, Japan

**Correction to: Molecular Neurobiology**

**https://doi.org/10.1007/s12035-023-03629-9**

The original version of this article unfortunately contained incorrect electronic supplementary material. With this, the unedited ESM was deleted.

The original article has been corrected.

