# Supplementary material for: Astrocytic Extracellular Vesicles Regulated by Microglial Inflammatory Responses Improve Stroke Recovery
Source: Mol Neurobiol. 2023 Sep 7;61(2):1002–21. doi: 10.1007/s12035-023-03629-9 (PMC10861643; doi:10.1007/s12035-023-03629-9)
Supplement: Supplementary file 1 — Supplementary Material 1 [file 12035_2023_3629_MOESM1_ESM.pdf]

SUPPLEMENTAL INFORMATION

Astrocytic extracellular vesicles regulated by microglial inflammatory responses improved stroke recovery

Chikage Kijima, MD[1]; Toshiki Inaba, PhD[1]; Kenichiro Hira, MD, PhD[1]; Nobukazu Miyamoto, MD, PhD[1]; Kazuo Yamashiro, MD, PhD[2]; Takao Urabe, MD, PhD[2]; Nobutaka Hattori, MD, PhD[1, 3], Yuji Ueno, MD, PhD[1]

1) Department of Neurology, Juntendo University Faculty of Medicine, Tokyo, Japan
2) Department of Neurology, Juntendo University Urayasu Hospital, Chiba, Japan
3) Neurodegenerative Disorders Collaborative Laboratory, RIKEN Center for Brain Science, Saitama, Japan

Supplementary Figures

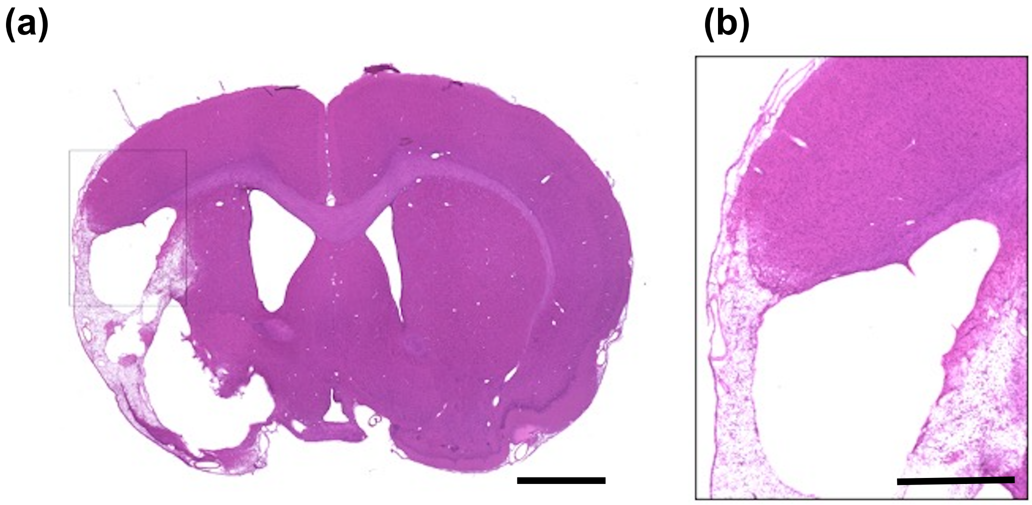

Supplementary Fig. S1. Peri-infarct area in chronic infarction

(a), (b). Image of brain coronal sections at 56 days after MCAO with Hematoxylin and Eosin staining, showing well demarcated infarction with cavitation in the territory supplied by MCA (A; B, magnified image). MCAO = Middle cerebral artery occlusion.

A. Scale bar = 2 mm, B. Scale bar = 1 mm

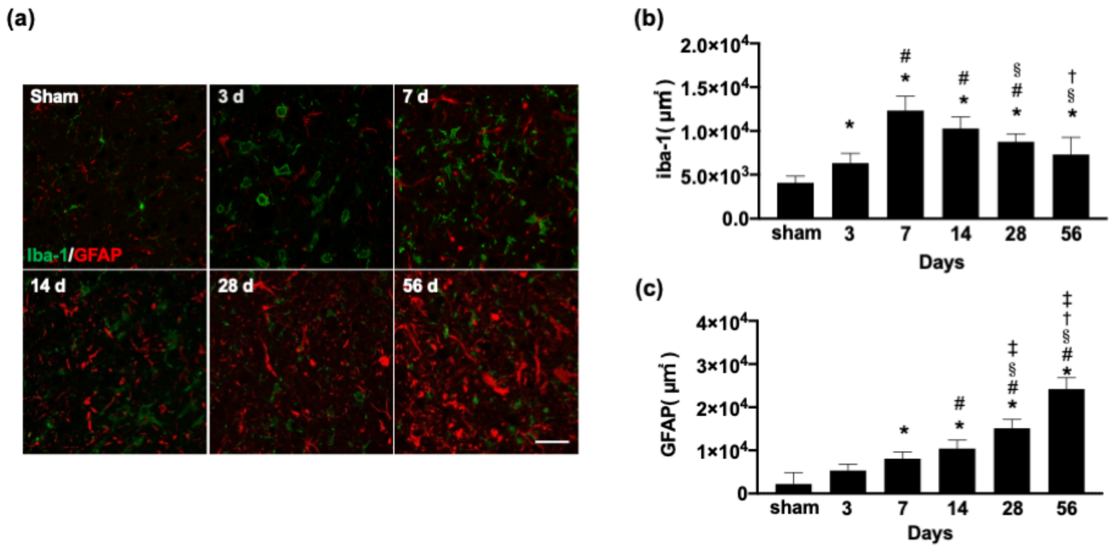

Supplementary Fig. S2. Temporal profile of Iba-1$^+$ and GFAP$^+$ area in the peri-infarct area.

(a). Double immunofluorescent confocal images of sham and the peri-infarct area at 3, 7, 14, 28, and 56 days after MCAO showing Iba-1$^+$ area (green) and GFAP$^+$ area (red). Scale bar = 50μm. **(b), (c).** Quantitative data on the area of Iba-1$^+$ area (**b**) and GFAP$^+$ area (**c**) in the peri-infarct area. Values are the mean ± SD. $^*P<0.05$ vs. sham, $^{\#}P<0.05$ vs. day 3, $^{\S}P<0.05$ vs. day 7, $^{\dagger}P<0.05$ vs. day 14, $^{\ddagger}P<0.05$ vs. day 28. N = 5/group (three sections per rat, and total of 15 samples in each group). Iba-1 = Ionized calcium-binding adapter molecule 1, GFAP = glial fibrillary acidic protein, MCAO = middle cerebral artery occlusion.

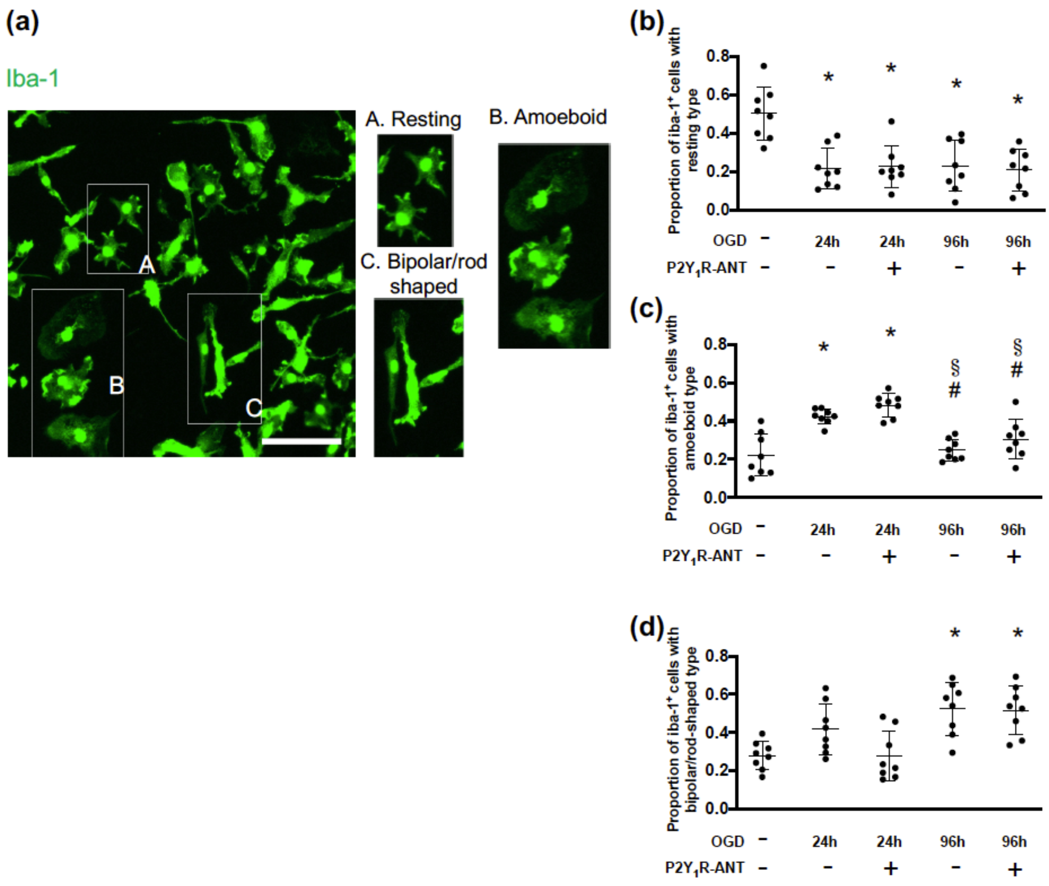

Supplementary Fig. S3. Morphology of cultured microglia after ischemia *in vitro*.

(a). Immunofluorescent confocal images of cultured microglia, showing resting (A), amoeboid (B), and bipolar/rod-shaped morphologies (C) of Iba-1^{+} microglia. Scale bar = 50 μm. (**b**)**, (c), (d).** Ratio of the number of cells of each cell morphology (**b**. resting type, **c**. amoeboid type, **d**. bipolar/rod-shaped) to the total number of Iba^{+} microglia before OGD, at 24 h after OGD, and at 96 h after OGD, and administration with $P2Y_1$R-ANT. N = 4/group. $^*P < 0.05$ vs. non-OGD microglia without $P2Y_1$R-ANT, $^{\#}P < 0.05$ vs. microglia at 24 h after OGD without $P2Y_1$R-ANT, $^{\S}P < 0.05$ vs. microglia at 24 h after OGD with $P2Y_1$R-ANT. Iba-1 = Ionized calcium-binding adapter molecule 1, OGD = oxygen-glucose deprivation, $P2Y_1$R-ANT = $P2Y_1$ receptor antagonist.

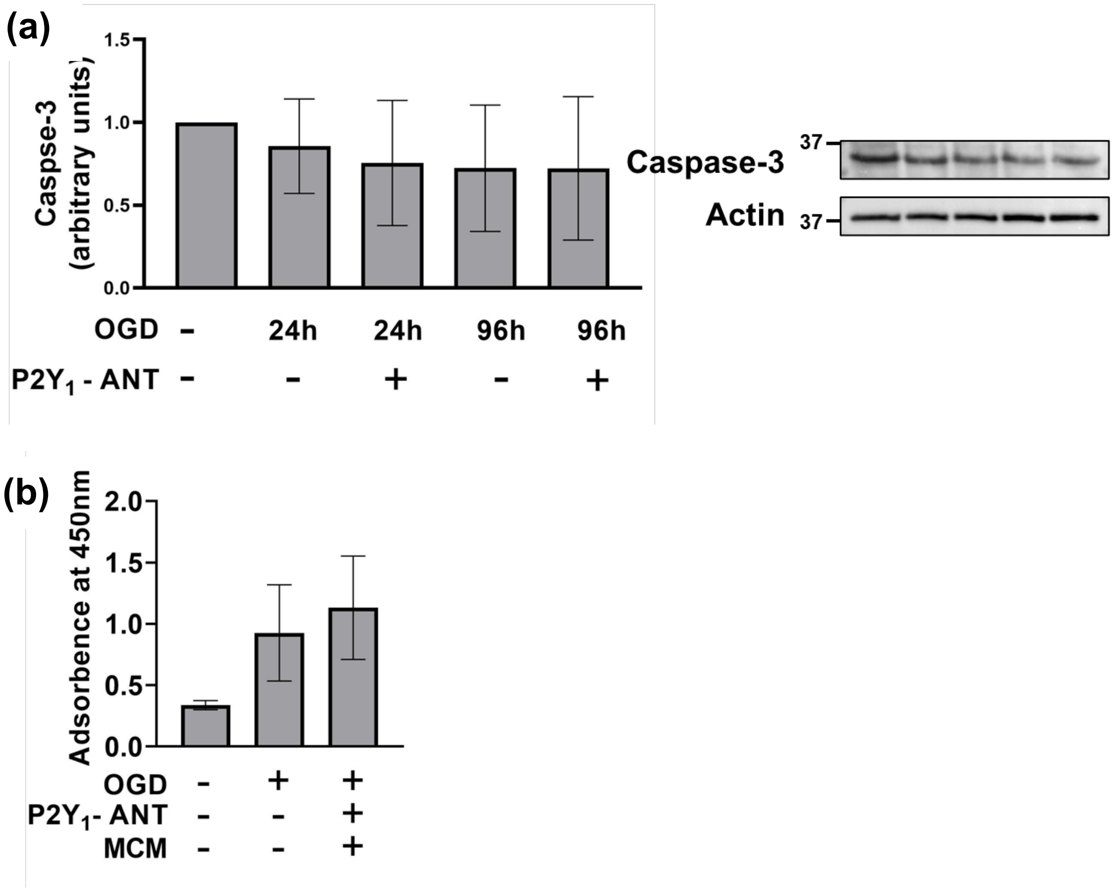

Supplementary Fig. S4. Cell survival of primary cultured microglia and astrocytes

(a). Western blots showing protein levels of caspase-3 in cultured microglia at 24 hours after OGD, at 24 hours after OGD with $P2Y_1R$- ANT administration, at 96 hours after OGD, and at 96 hours after OGD with $P2Y_1R$- ANT administration (0.86 ± 0.29, 0.75 ± 0.38, 0.72 ± 0.38, 0.72 ± 0.43 [arbitrary units]) relative to cultured microglia without OGD. N = 3/group. **(b).** Quantitative data of cell viability in non-OGD astrocytes, OGD astrocytes, OGD astrocytes treated with 1 mM $P2Y_1R$- ANT and MCM (0.34±0.04, 0.93±0.39, 1.13±0.42). N = 4/group. Values are the mean ± SD. OGD = oxygen-glucose deprivation, $P2Y_1R$- ANT = $P2Y_1$ receptor antagonist, MCM = microglia conditioned medium.

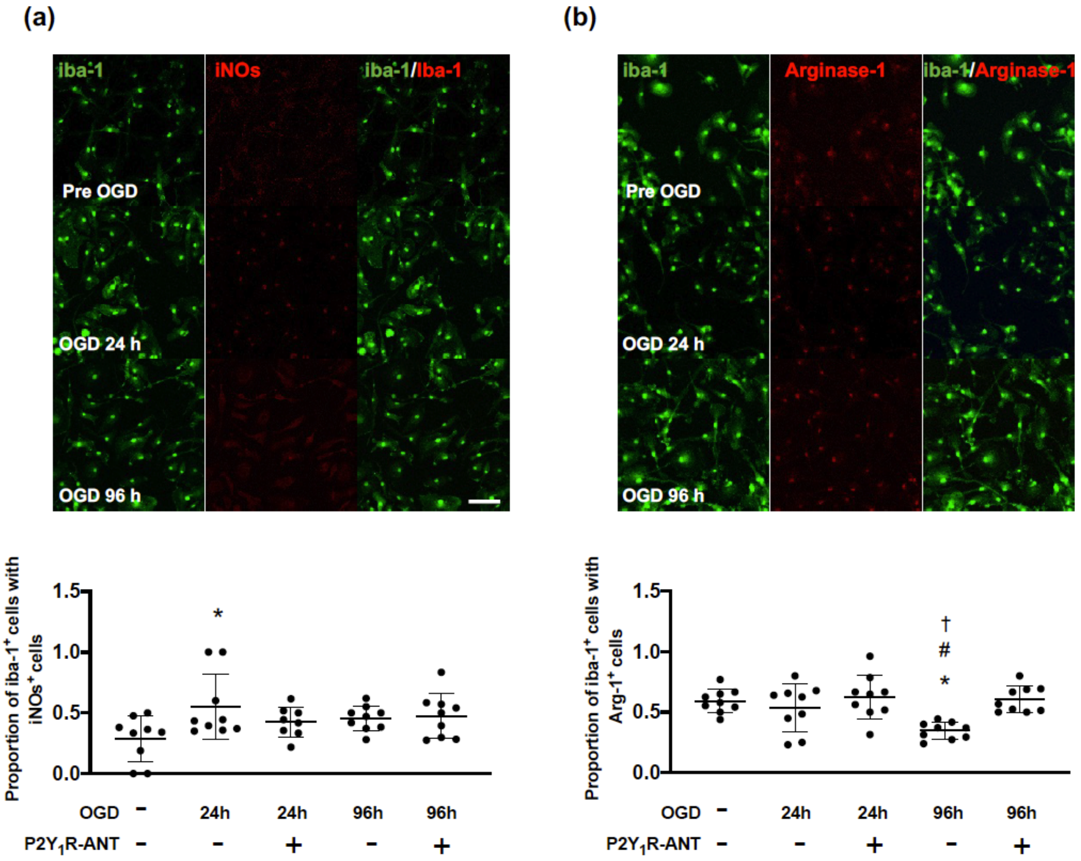

Supplementary Fig. S5. Alteration of M1/M2 phenotypes after ischemia *in vitro*.

(a), (b). Double immunofluorescent confocal images of cultured microglia showing iNOS$^+$ cells (red) (**a**) and arginase-1$^+$ cells (red) (**b**), and Iba-1$^+$ cells (green). Scale bar = 50 μm. Ratio of iNOS$^+$ cells and arginase-1$^+$ cells to total Iba-1$^+$ cells before OGD, at 24 h after OGD, at 96 h after OGD, and after $P2Y_1$R-ANT (1 mM) administration after OGD. N = 4/group. $^*P < 0.05$, vs. non-OGD microglia without $P2Y_1$R-ANT, $^\#P < 0.05$ vs. microglia at 24 h after OGD with $P2Y_1$R-ANT, $^\dagger P < 0.05$ vs. microglia at 96 h after OGD with $P2Y_1$ receptor antagonist. Iba-1 = Ionized calcium-binding adapter molecule 1, iNOS = inducible nitric oxide synthase, OGD = oxygen-glucose deprivation, $P2Y_1$R-ANT = $P2Y_1$ receptor antagonist.

(a)

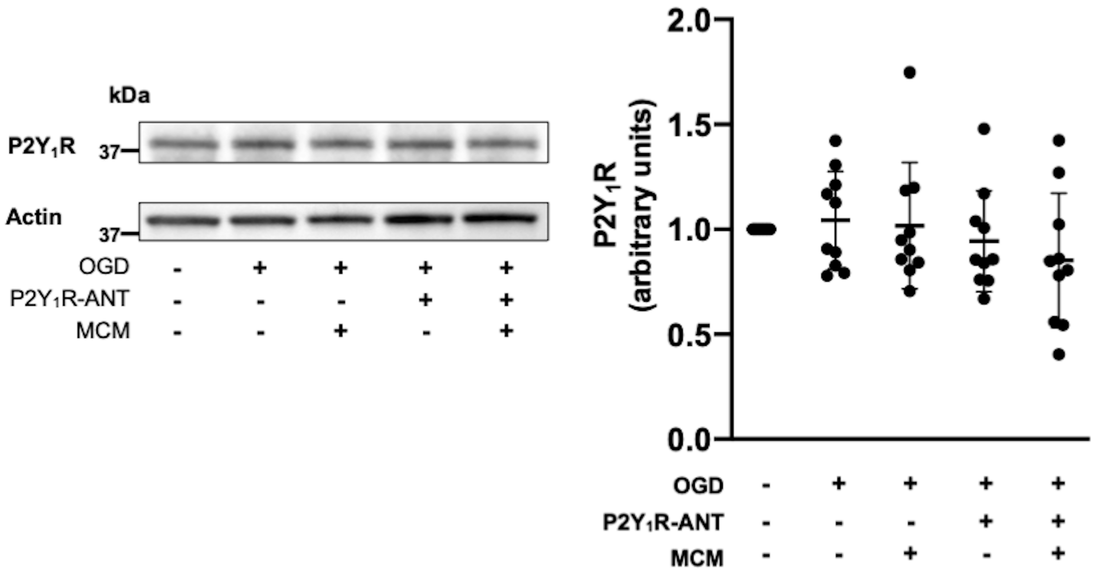

Supplementary Fig. S6. Expression of $P2Y_1R$ in cultured astrocytes

(a). Western blots showing protein levels of $P2Y_1$ receptor in non-OGD astrocytes, OGD astrocytes, OGD astrocytes treated with MCM, OGD astrocytes treated with 1 mM $P2Y_1R$-ANT, and OGD astrocytes treated with $P2Y_1R$- ANT and MCM. β-actin were used as internal controls. N = 5/group. Values are the mean ± SD. OGD = oxygen-glucose deprivation, MCM = microglia conditioned medium, $P2Y_1R$-ANT = $P2Y_1$ receptor antagonist.

(a)

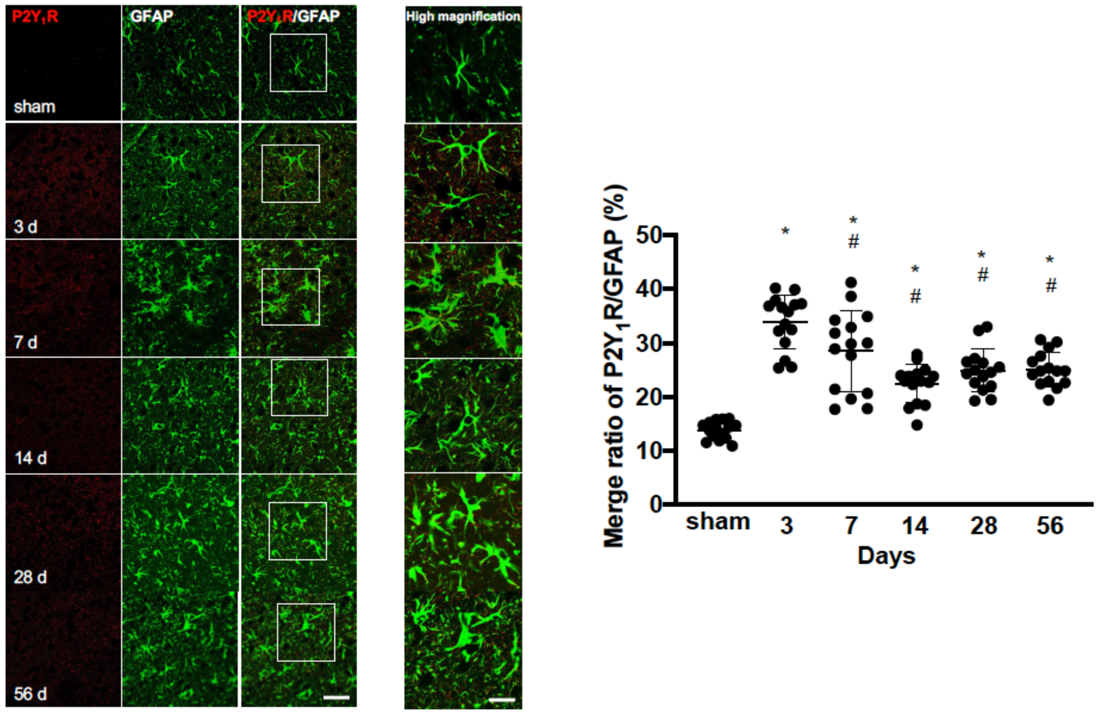

Supplementary Fig. S7. Temporal profile of $P2Y_1R^+$ and $GFAP^+$ area in the peri-infarct area.

(a). Double immunofluorescent confocal images of sham and the peri-infarct area at 3, 7, 14, 28, and 56 days after MCAO showing $P2Y_1R^+$ (red) and $GFAP^+$ area (green). Scale bar = 100 µm, and 50 µm in high-magnification image. Quantitative data on the area of $P2Y_1R^+GFAP^+$ area in the peri-infarct area. Values are the mean ± SD. *P<0.05 vs. sham, $^{\#}P$<0.05 vs. day 3, N = 5/group (three sections per rat, and total of 15 samples in each group). $P2Y_1R$ = $P2Y_1$ receptor, GFAP = glial fibrillary acidic protein, MCAO = middle cerebral artery occlusion.

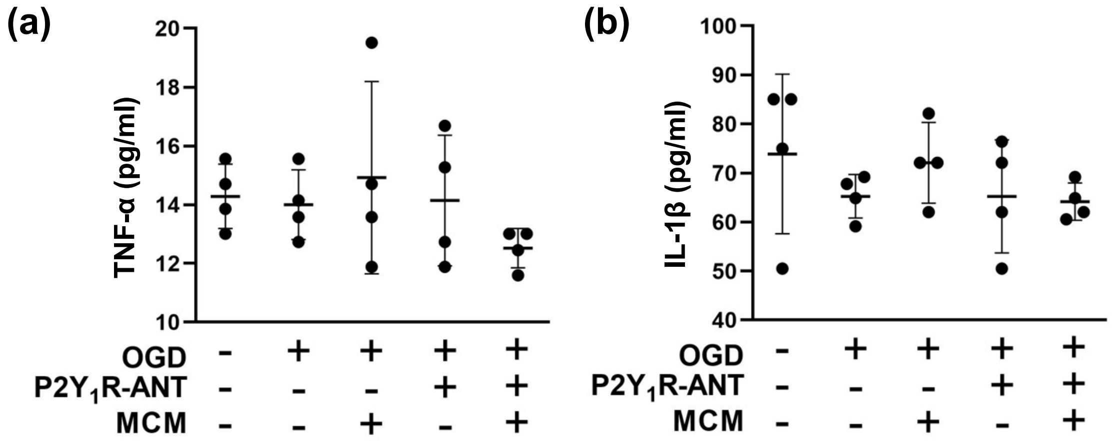

Supplementary Fig. S8. TNF-α and IL-1β levels in astrocyte-conditioned media

(a), (b). ELISA showing TNF-α and IL-1β levels in non-OGD astrocytes, OGD astrocytes, OGD astrocytes treated with MCM, OGD astrocytes treated with 1 mM $P2Y_1R$- ANT, and OGD astrocytes treated with $P2Y_1R$- ANT and MCM. N = 4/group. Values are the mean ± SD. TNF-α = tumor necrosis factor-α, IL-1β = ianterleukin-1β, ELISA = enzyme-linked immunosorbent assay OGD = oxygen-glucose deprivation, MCM = microglia conditioned medium, $P2Y_1R$-ANT = $P2Y_1$ receptor antagonist.

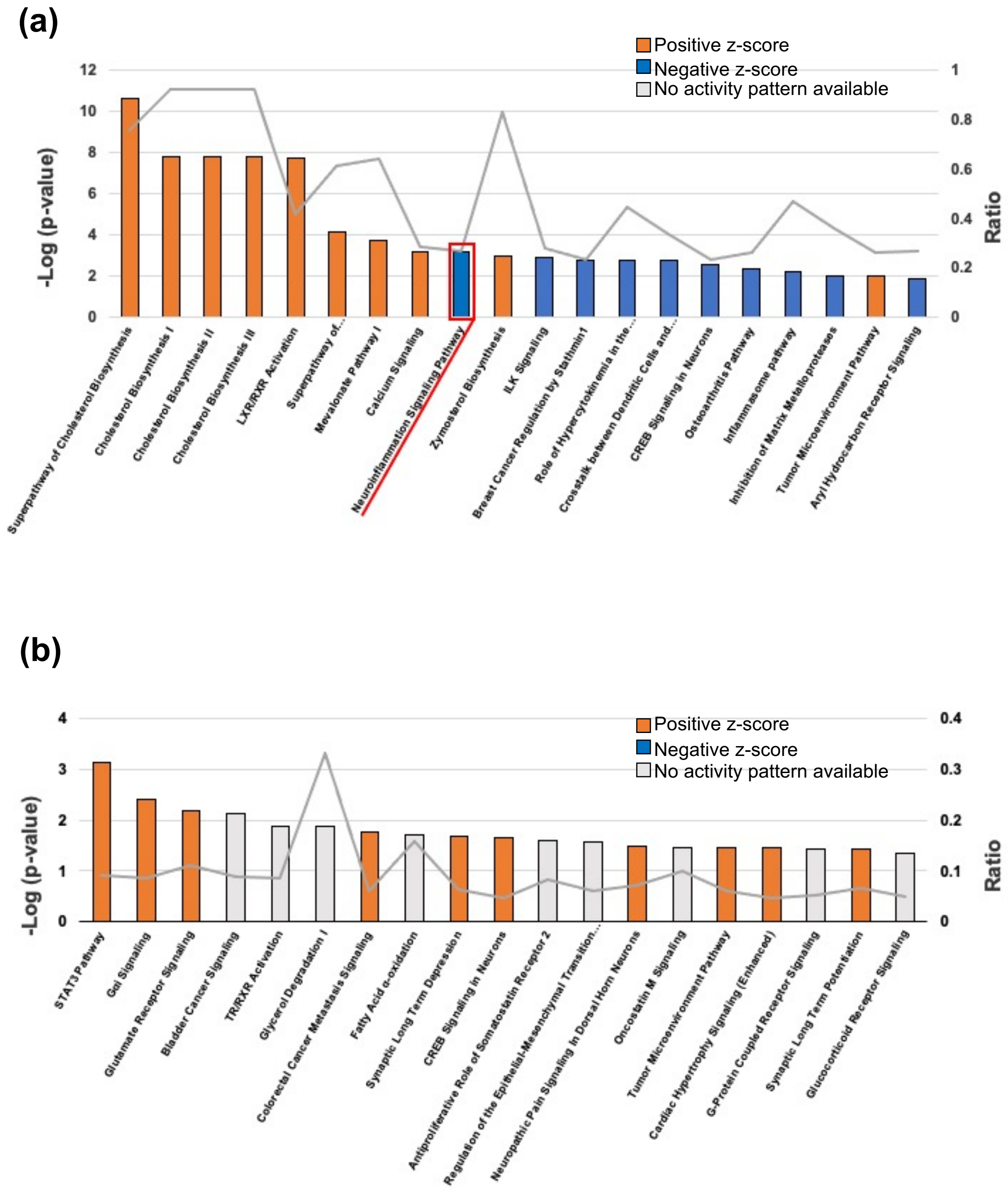

Supplementary Fig. S9. Significant canonical pathways for each treatment in ischemic astrocytes

(a), (b). Top 20 canonical pathways by IPA "Core analysis" for OGD astrocytes treated with $P2Y_1$R-ANT and MCM relative to OGD astrocytes treated with $P2Y_1$R-ANT (a) and OGD astrocytes treated with $P2Y_1$R-ANT relative to OGD astrocytes (b). Statistically significant canonical pathways are listed according to their p value (−log) ≥1.3. “Ratio” indicates the number of molecules from the data set that map to the pathway listed divided by the total

number of molecules that map to the canonical pathway from within the IPA database. Blue bars: negative z-score; orange bars: positive z-score; gray bars: no activity pattern available; white bars: activity of zero. IPA = Ingenuity Pathway Analysis (IPA), OGD = oxygen-glucose deprivation, $P2Y_1R$- ANT = $P2Y_1$ receptor antagonist, MCM = microglia conditioned medium.

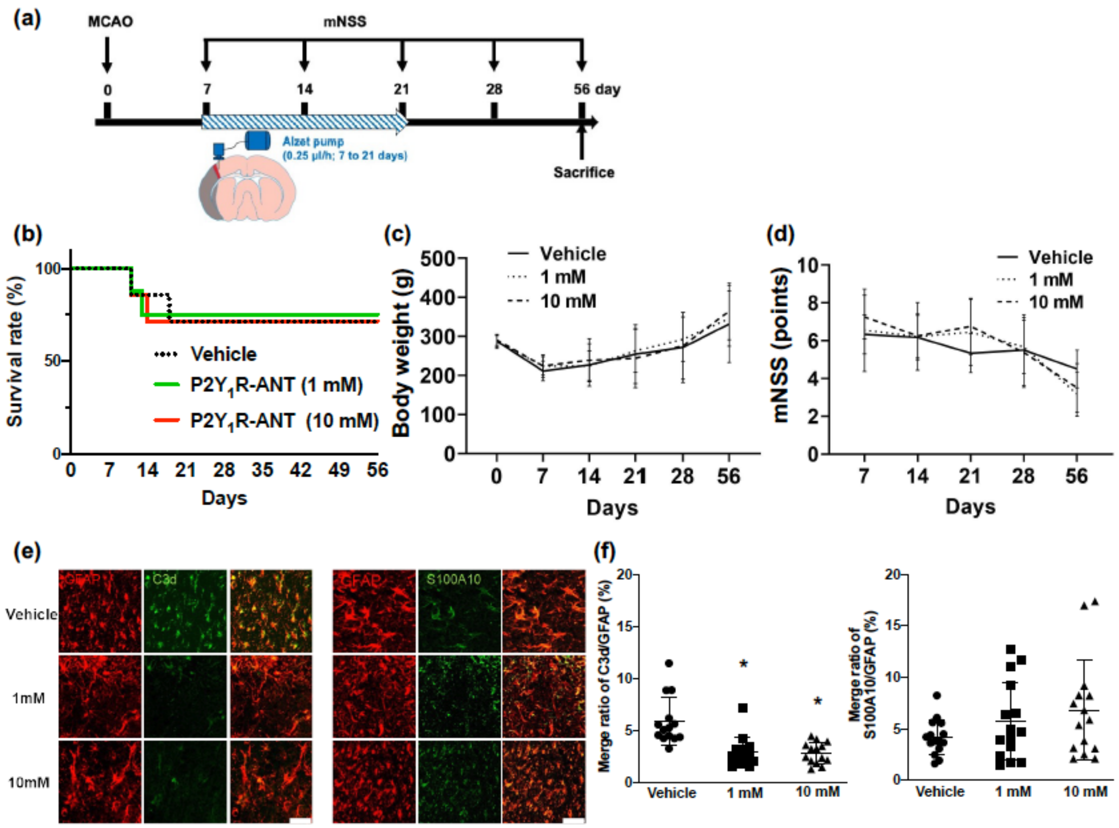

Supplementary Fig. S10. Therapeutic effect of P2Y$_1$R-ANT on the peri-infarct area after MCAO.

(a). The experimental scheme of MCAO and treatment with P2Y$_1$R-ANT. **(b), (c), (d).** Survival rate, N =13/group (**b**), body weight, N = 6-9/group (**c**), and modified neurological severity score N = 6/group (**d**) in PBS-treated, and 1 mM and 10 mM of P2Y$_1$R-ANT-treated rats subjected to MCAO. **(e).** Double immunofluorescent confocal images of the peri-infarct area at 56 days after MCAO with intracerebral administration of PBS (vehicle), 1 mM and 10 mM P2Y$_1$R-ANT showing GFAP$^+$ area (red), and C3d$^+$ area and S100A10$^+$ area (green). Scale bar = 100 µm. **(f).** Merge ratio of C3d/GFAP and S100A10/GFAP. N = 5/group (three sections per rat, and total of 15 samples in each group). Values are the mean ± SD. $^*P$<0.05 vs. PBS-treated rats. P2Y$_1$R-ANT = P2Y$_1$ receptor antagonist, MCAO = middle cerebral artery occlusion, GFAP = glial fibrillary acidic protein.

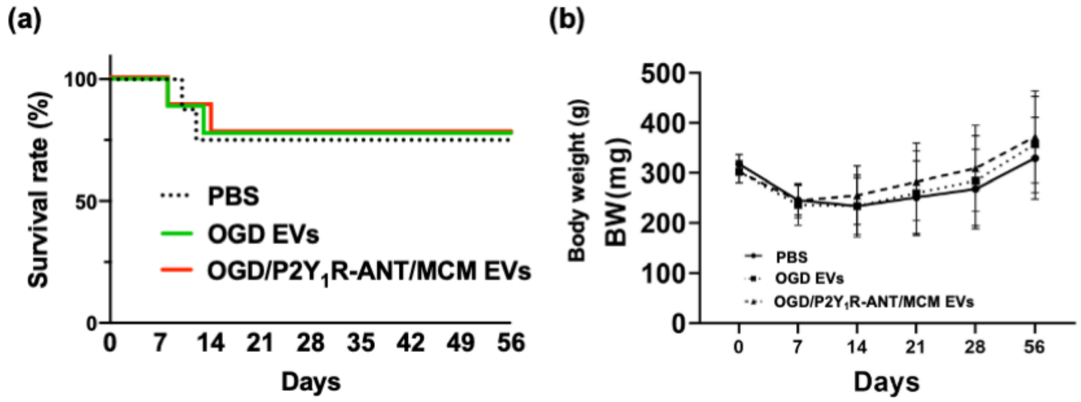

Supplementary Fig. S11. Survival rate and body weight after treatment with AEVs derived from anti-inflammatory astrocytes after MCAO.

(a), (b). Survival rate, N =11-13/group (**a**) and body weight, N = 6-7/group (**b**) in PBS-treated, and treatment with AEVs derived from OGD astrocytes (100 μg) and OGD astrocytes treated with $P2Y_1R$-ANT and MCM (100 μg) rats subjected to MCAO. AEVs = astrocytic extracellular vesicles, MCAO = middle cerebral artery occlusion, $P2Y_1R$-ANT = $P2Y_1$ receptor antagonist, MCM = microglia conditioned medium, GFAP = glial fibrillary acidic protein.

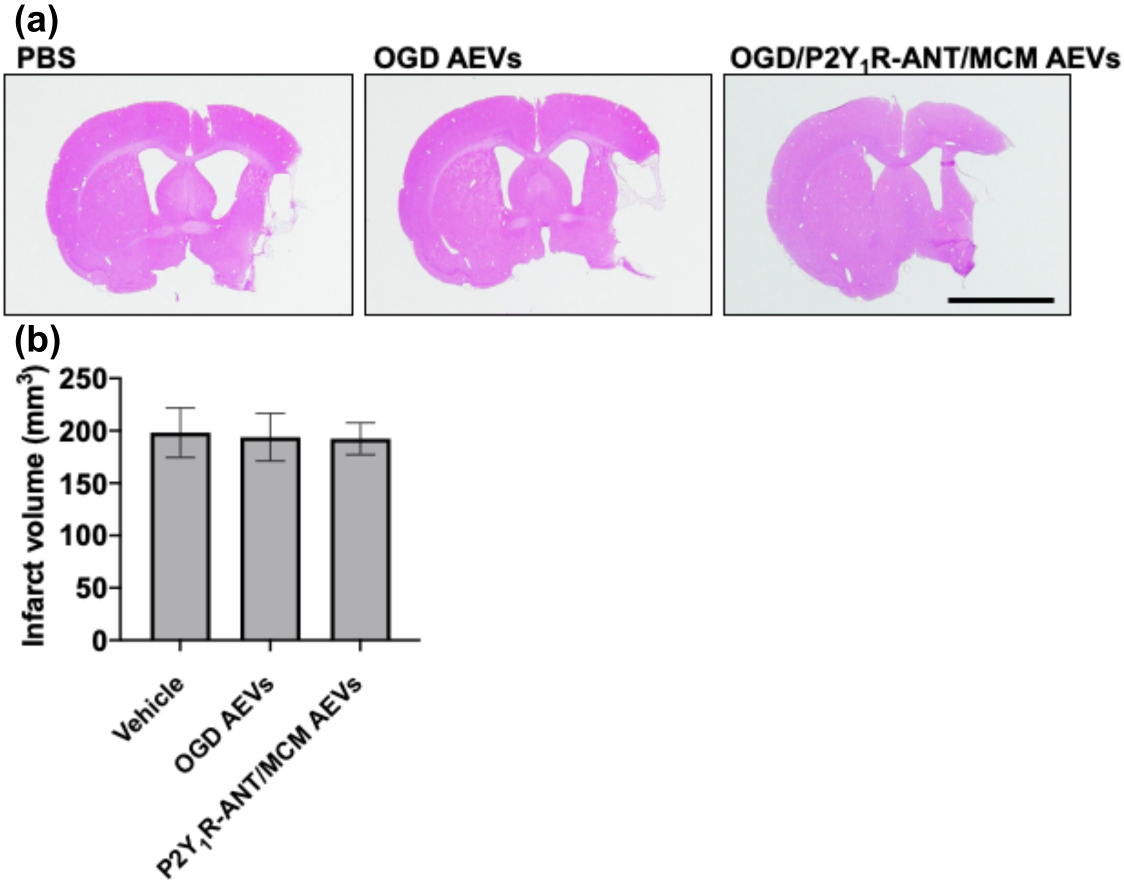

Supplementary Fig. S12. Infarct volume after AEVs treatment in MCAO rats

(a). Representative images of brain coronal sections stained with Hematoxylin and Eosin staining from the PBS-treated rats, and rats treated with AEVs derived from OGD astrocytes and OGD astrocytes treated with $P2Y_1$R- ANT and MCM, showing infarct volumes at 56 days after MCAO. **(b).** Quantitative data of infarct volumes in the PBS-treated rats, and rats treated with AEVs derived from OGD astrocytes and OGD astrocytes treated with $P2Y_1$R- ANT and MCM. N = 3/group. Values are the mean ± SD. AEVs = astrocytic extracellular vesicles, MCAO = middle cerebral artery occlusion, OGD = oxygen-glucose deprivation, $P2Y_1$R-ANT = $P2Y_1$ receptor antagonist, MCM = microglia conditioned medium. Scale bar: 5 mm. Scale bar = 5 mm

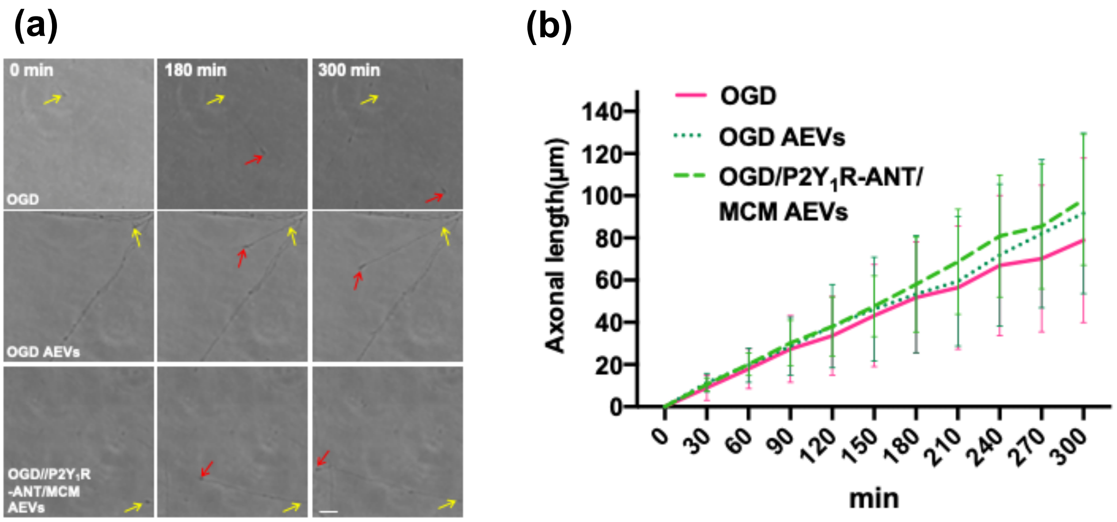

Supplementary Fig. S13. Axonal elongation after AEVs treatment

(a). Representative time-lapse microscopic images of primary cortical neurons in a microfluidic chamber showing axonal elongation (distance from yellow arrow to red arrow) in OGD neurons, OGD neurons treated with AEVs derived from OGD astrocytes, and OGD neurons derived from AEVs derived from OGD astrocytes treated with $P2Y_1R$-ANT and MCM. Scale bar = 20 μm. **(b).** Quantitative data of axonal elongation per 30 min prior to 96 h after OGD. N = 3/group. Values are the mean ± SD. AEVs = astrocytic extracellular vesicles, OGD = oxygen-glucose deprivation, $P2Y_1R$-ANT = $P2Y_1$ receptor antagonist, MCM = microglia conditioned medium, pNFH = phosphorylated neurofilament heavy chain.

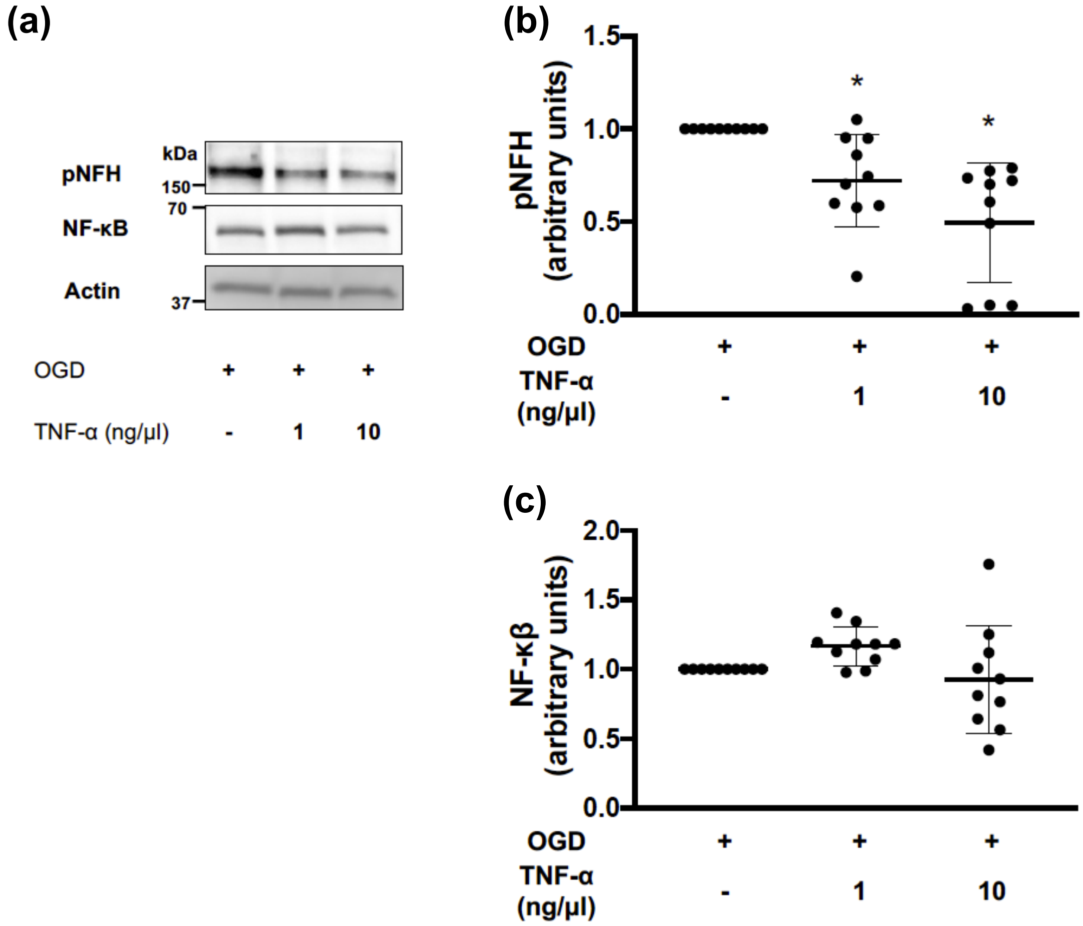

Supplementary Fig. S14. pNFH and NF-κB **expression after TNF-α treatment**

(a), (b), (c). Representative western blots showing protein levels of pNFH and NF-κB (**a**) and quantitative data of pNFH (**b**) and NF-κB (**c**) in OGD neurons, and OGD neurons treated with 1 and 10 ng/μL of TNF-α. N = 4-5/group. $^{*}P$<0.05 vs. OGD neurons. OGD = oxygen-glucose deprivation, pNFH = phosphorylated neurofilament heavy chain, NF-κB = nuclear factor-κβ, TNF-α= tumor necrosis factor-α.

Supplementary Table 1. Categories and microRNAs of Inflammatory Reactions in Top Diseases and functions of IPA

Categories	Diseases or Functions Annotation	Molecules	p-value
Inflammatory Response,Organismal Injury and Abnormalities	Inflammation of organ	miR-124-3p (and other miRNAs w/seed AAGGCAC),miR-130a-3p (and other miRNAs w/seed AGUGCAA),miR-133a-3p (and other miRNAs w/seed UUGGUCC),miR-146a-5p (and other miRNAs w/seed GAGAACU),miR-208a-3p (and other miRNAs w/seed UAAGACG),miR-3118 (and other miRNAs w/seed GUGACUG),miR-346 (and other miRNAs w/seed GUCUGCC),miR-501-5p (miRNAs w/seed AUCCUUU),miR-532-5p (and other miRNAs w/seed AUGCCUU),miR-92a-3p (and other miRNAs w/seed AUUGCAC)	0.000108
Inflammatory Response	Inflammation of body cavity	miR-124-3p (and other miRNAs w/seed AAGGCAC),miR-130a-3p (and other miRNAs w/seed AGUGCAA),miR-133a-3p (and other miRNAs w/seed UUGGUCC),miR-208a-3p (and other miRNAs w/seed UAAGACG),miR-3118 (and other miRNAs w/seed GUGACUG),miR-346 (and other miRNAs w/seed GUCUGCC),miR-501-5p (miRNAs w/seed AUCCUUU),miR-532-5p (and other miRNAs w/seed AUGCCUU),miR-92a-3p (and other miRNAs w/seed AUUGCAC)	0.0000572
Inflammatory Response	Inflammation of absolute anatomical region	miR-124-3p (and other miRNAs w/seed AAGGCAC),miR-130a-3p (and other miRNAs w/seed AGUGCAA),miR-133a-3p (and other miRNAs w/seed UUGGUCC),miR-208a-3p (and other miRNAs w/seed UAAGACG),miR-3118 (and other miRNAs w/seed GUGACUG),miR-346 (and other miRNAs w/seed GUCUGCC),miR-501-5p (miRNAs w/seed AUCCUUU),miR-532-5p (and other miRNAs w/seed AUGCCUU),miR-92a-3p (and other miRNAs w/seed AUUGCAC)	0.000227
Inflammatory Disease,Inflammatory Response,Organismal Injury and Abnormalities,Renal and Urological Disease	Class II lupus nephritis	miR-124-3p (and other miRNAs w/seed AAGGCAC),miR-130a-3p (and other miRNAs w/seed AGUGCAA),miR-133a-3p (and other miRNAs w/seed UUGGUCC),miR-208a-3p (and other miRNAs w/seed UAAGACG),miR-3118 (and other miRNAs w/seed GUGACUG),miR-346 (and other miRNAs w/seed GUCUGCC),miR-92a-3p (and other miRNAs w/seed AUUGCAC)	3.84E-11
Immunological Disease,Inflammatory Disease,Inflammatory Response,Neurological Disease,Organismal Injury and Abnormalities	Experimental autoimmune encephalomyelitis	miR-124-3p (and other miRNAs w/seed AAGGCAC),miR-133a-3p (and other miRNAs w/seed UUGGUCC),miR-3118 (and other miRNAs w/seed GUGACUG),miR-346 (and other miRNAs w/seed GUCUGCC),miR-92a-3p (and other miRNAs w/seed AUUGCAC)	0.000223
Gastrointestinal Disease,Inflammatory Disease,Inflammatory Response,Organismal Injury and Abnormalities	Ulcerative colitis	miR-501-5p (miRNAs w/seed AUCCUUU),miR-532-5p (and other miRNAs w/seed AUGCCUU)	0.0448
Cellular Movement,Hematological System Development and Function,Immune Cell Trafficking,Inflammatory Response	Migration of monocytes	miR-133a-3p (and other miRNAs w/seed UUGGUCC),miR-146a-5p (and other miRNAs w/seed GAGAACU)	0.0069
Cellular Movement,Hematological System Development and Function,Immune Cell Trafficking,Inflammatory Response	Migration of neutrophils	miR-133a-3p (and other miRNAs w/seed UUGGUCC),miR-146a-5p (and other miRNAs w/seed GAGAACU)	0.0155
Cell-To-Cell Signaling and Interaction,Hematological System Development and Function,Immune Cell Trafficking,Inflammatory Response	Activation of bone marrow-derived macrophages	miR-124-3p (and other miRNAs w/seed AAGGCAC)	0.0239
Cell-To-Cell Signaling and Interaction,Cellular Function and Maintenance,Inflammatory Response	Phagocytosis by dendritic cells	miR-142-3p (and other miRNAs w/seed GUAGUGU)	0.0163
Cell-To-Cell Signaling and Interaction,Cellular Function and Maintenance,Inflammatory Response	Phagocytosis of E. coli	miR-142-3p (and other miRNAs w/seed GUAGUGU)	0.0313
